# Internet-facilitated interventions for informal caregivers of patients with neurodegenerative disorders: Systematic review and meta-analysis

**DOI:** 10.1177/20552076221129069

**Published:** 2022-10-20

**Authors:** Neil Boyt, Aileen K Ho, Hannah Morris-Bankole, Jacqueline Sin

**Affiliations:** 1 School of Psychology & Clinical Language Sciences, University of Reading, Earley Gate, RG6 6AL, England; 2 School of Health Sciences, University of London, Myddelton Street Building, Myddelton Street, London, EC1R 1UW, England

**Keywords:** digital, general, internet, general, informal caregivers, neurodegenerative disorders, dementia, disease, Alzheimer disease, online, general, web-based, systematic review, carer

## Abstract

**Objective:**

This systematic review explored the effectiveness of internet-delivered interventions in improving psychological outcomes of informal caregivers for neurodegenerative-disorder (ND) patients.

**Methods:**

We searched seven databases for English-language papers published from 1999 to May 2021. Study-eligibility required that interventions used a minimum 50% internet-facilitation, targeting unpaid, adult informal caregivers of community-based ND-patients. We included randomised controlled trials (RCTs) and pre-post evaluative studies reporting outcomes for at least one-time point post-intervention. Independent quality checks on abstract and full-text screening were completed. Data extraction encompassed interventions’ features, approaches, theoretical bases and delivery-modes. The Integrated quality Criteria for the Review Of Multiple Study designs (ICROMS) framework assessed risk of bias. Alongside narrative synthesis, we calculated meta-analyses on post-intervention using outcome measures from at least two RCTs to assess effectiveness.

**Results:**

Searches yielded 51 eligible studies with 3180 participants. In 48 studies, caregivers supported a dementia-diagnosed individual. Intervention-durations encompassed four weeks to 12 months, with usage-frequency either prescribed or participant-determined. The most frequently-used approach was education, followed by social support. We calculated meta-analyses using data from 16 RCTs. Internet-delivered interventions were superior in improving mastery (g  =  1.17 [95% CI; 0.1 to 2.24], p  =  0.03) and reducing anxiety (g = -1.29 [95% CI; −1.56 to −1.01], p < 0.01), compared to all controls. Findings were equivocal for caregivers’ quality of life, burden and other outcomes. High heterogeneity reflected the multifarious combinations of approaches and delivery-modes, precluding assessment of the most efficacious intervention features. Analyses using burden and self-efficacy outcomes’ follow-up data were also non-significant compared to all comparator-types. Although 32 studies met the ICROMS threshold scores, we rated most studies’ evidence quality as ‘very-low’.

**Conclusions:**

This review demonstrated some evidence for the efficacy of internet-delivered interventions targeting informal ND-caregivers. However, more rigorous studies, with longer follow-ups across outcomes and involving NDs other than dementia, are imperative to enhance the knowledge-base.

## Introduction

Neurodegenerative disorders (ND) present an increasing health exigency worldwide^1^ being the foremost cause of increases in disability and second highest cause of death.^2^ Furthermore, the prevalence rate of the more common NDs such as Alzheimer's disease (AD) and Parkinson's disease (PD) is predicted to double in the next 20 years.^1^ Rarer NDs such as Huntington disease's (HD),^3^ amyotrophic lateral sclerosis,^4^ or multiple sclerosis (MS)^5^ have a devastating impact on patient and carer health and quality of life.

These NDs are relentlessly progressive with no known cure. Patients typically experience a complex, variable and unpredictable course of disease^6^ over years and often decades. Changes in behavioural, cognitive and motor skills^7^ affect instrumental (e.g. managing money, transportation, etc.) and basic activities of daily living (e.g. feeding, dressing and washing)^8–10^ escalating caregiver burden.^11,12^ ND care-provision is distinctly demanding^13^ and is associated with sleep impairment,^14^ anxiety,^15,16^ stress,^17^ depression,^18,19^ cognitive decline^20^ increased heart-disease risk^21^ and exacerbation of pre-existing conditions.^22^

Caregivers risk experiencing burnout. Where care-provision becomes both nonviable and deleterious^23^ the decision to institutionalise care-recipients is inexorable.^24^ To address this, different caregiver interventions such as psychoeducation,^25^ counselling,^26^ cognitive behavioral therapy^27^ and support groups^28^ are promising, but their effects vary. These studies are affected by low engagement and high attrition rates^29^ often due to the complexity, time demands and intensity of care-provision.^30^

Internet-facilitated interventions have improved access through being flexible and cost-effective.^31^ This is further enhanced by the recent proliferation in smartphone ownership, concomitant with availability of mobile applications.^32–34^ Traditionally face-to-face interventions successfully transferred online include videoconferencing-based clinical visits^35^ particularly during the recent Covid-19 pandemic-enforced lockdowns,^36^ web-based support groups^37^ and psychosocial interventions for caregiving dyads.^38^ Caregivers are also able to access support networks through social media platforms^39,40^ whilst connecting with caregiver-peers.^37^

Most reviews of caregiver interventions focus on dementia patients.^41^ These reviews highlight the plurality of intervention characteristics and features,^42–43^ psychological constructs targeted^44^ and delivery modalities employed. Although there is some agreement that multicomponent interventions (e.g. psychoeducation with CBT) are more effective in improving caregiver outcomes,^42,45^ consensus around efficacious components^46^ or underlying mechanisms^47^ remains elusive. Additionally, there appears to be an absence of literature on internet-based interventions in less common NDs, such as PD,^22^ amyotrophic lateral sclerosis (ALS) and rare NDs, for example, HD.^48^

This review investigated the efficacy of internet-facilitated interventions are at improving well-being and other health outcomes for informal caregivers of community-based, ND-diagnosed individuals. Four specific questions were addressed: (1) What therapeutic approaches and theoretical bases are most frequently used in interventions? (2) Which features are common within internet-facilitated interventions’ content and design? (3) How effective are interventions in promoting informal caregivers’ psychological health when compared to standard care alone or other active comparators (e.g. face-to-face)? (4) Which intervention approach and design feature (e.g. group vs. individual) is most efficacious?

## Methods

This study was prospectively registered on Prospero before searches started^49^ and was conducted in accordance with the latest PRISMA guidelines.^50^ A completed checklist can be found in Appendix 1.

### Search strategy and data sources

We searched the following databases, from 1999 until May 2021, for published studies written in English: MEDLINE and MEDLINE in-process (via Ovid); PsycINFO (via Ovid), NIH Clinical Trials; Cochrane Central Register of Controlled Trials (CENTRAL); Web of Science and PubMed. Following establishment of a provisional list of studies, their citation indexes were checked for any similar studies not captured by the initial search,^51^ alongside hand-searching the reference lists of other reviews covering analogous intervention-types (e.g. internet-facilitated interventions for unpaid caregivers of patients with similar conditions).

A search strategy using key search terms compiled through a combination of the study authors’ (AH, JS) expertise and consultation of the extant literature was formulated. Terms were devised using the Population (e.g. caregiv*/neurodegener*/informal); Intervention (iPhone/digital/technology); Comparison intervention and Outcome measures (PICO) framework,^52^ concentrating on population and intervention terms, to ensure a highly sensitive search. Searches were executed using a mixture of entered keywords and existing Medical Subject Heading terms (MeSH), which were ‘exploded’ to encompass related terms. Truncation conventions, common abbreviations and Boolean operators were utilised where appropriate in order to optimise the search's sensitivity. For each database, the search strategy was customised. A complete list of search terms for each database are detailed in Appendix 2.

### Study eligibility criteria and selection

Studies included adult individuals (18 years-old + ) who provide unpaid care to another community-based person with a neurodegenerative disease (ND) diagnosis. These included AD and related Dementias (e.g. frontotemporal dementia; dementia with Lewy bodies), MS, PD, motor neuron disease (e.g. amyotrophic lateral sclerosis) and HD for the latter this encompassed care-recipients with either a family history of or a positive genetic test for HD. In caregiver-recipient dyad studies, the reporting of outcome-measures exclusively derived from caregivers were required. We excluded studies involving formal, professional or paid caregivers, alongside institutionalised care-provision. Eligible caregiver-targeted interventions involved at least 50% delivery via an internet-facilitated device (e.g. smartphone, desktop computer). Any interventions that employed non-internet facilitated storage-media (e.g. CD-ROM, DVD's) only alongside those that were solely focused on practical skills training (e.g. lifting, bathing) were excluded. Where studies utilised a comparator, it was categorised as either: 1) Inactive: Wait-list/usual care/no treatment/non-active technological comparison (e.g. read-only information delivered via storage media); or 2) Active: non-technological active comparison (e.g. face-to-face delivered therapy or carer support group).

After downloading search results from all databases into an EndNote Online library, duplicates were removed using both the ‘deduplication’ function and manual identification. NB implemented initial screening of all titles and abstracts. For studies deemed either apposite or requiring further information, NB administered full-text screening. These stages were carried out independently by HBM, who screened at least 20% of the titles and abstracts, and full-text studies, in parallel. Disagreements between screenings were resolved through team discussion.

For inclusion in this review, studies needed to be quantitative studies reporting data from validated outcome measures capturing changes in psychological health variables (e.g. burden, perceived quality of life (PQoL) or health-related quality of life (HRQoL)). We included both controlled and non-controlled before-after studies (e.g. a single intervention group to test feasibility) in addition to randomized controlled trials. Correspondingly, this excluded protocol or other descriptive studies that did not provide outcome data. For estimating effect measures, only data reported by RCT's were considered in evaluating intervention effectiveness.

### Data extraction

Relevant information from eligible full-text papers was recorded on data extraction forms, designed through adaptation of the Cochrane Handbook of Systematic Reviews^52^ guidelines to the specific objectives of this study (blank example in Appendix 4). Data extracted included; study setting, sample size in each arm, study participants’ characteristics and patient's ND diagnosis. We recorded intervention features such as; intervention's duration, delivery intensity (e.g. weekly) and features (e.g. videoconferencing), group versus individual participation and features of comparator conditions. Alongside the outcome measures used, post-intervention group means and standard deviations were extracted, with post-intervention and any follow-up data recorded separately.

In order to address review questions regarding the most frequently employed approaches, Chi & Demiris’^54^ classification system to categorise interventions was incorporated as follows:

1. Education (e.g. educational websites or videos); 2) Consultation; 3) Social support (e.g. peer group meetings); 4) Psychosocial/cognitive behavioural therapy (remotely delivered therapy, coaching); 5) Data collection and monitoring systems (e.g. experience sampling method (ESM)); and 6) Clinical care delivery (e.g. therapy delivered to caregiver & patient over videoconferencing).

To investigate the theoretical bases used in intervention design and implementation, we administered elements of Michie & Prestwich's^55^ theory coding scheme (TCS), applying the eleven items that identify how theory and predictors or constructs are assimilated into interventions’ framework. Items 1–6 are applicable where interventions studies either mention theories or predictors, base selection of participants on them, or use them to select, develop or tailor techniques. Items 7–11 concern the linking of intervention-techniques to theory, constructs or predictors. Items 12–19 were not utilised, as these were focused on construct measurement, mediation effects and whether outcomes led to theory alteration. Because our analysis was for classification purposes only, we opted not to employ their scoring system.

To investigate the modes of delivery used in intervention design and implementation, Webb et al.'s^56^ coding convention was applied in which one or more of the following categories could be assigned:

1. *Automated functions*. i) providing an enriched environment (e.g. access to content and other links); ii) providing tailored feedback based on monitoring (reinforcement messaging); iii) follow-up messaging (e.g. reminders, encouragement)
2. *Communicative functions.* i) scheduled contact with an advisor (e.g. emails); ii) access to an advisor to receive advice (e.g. ‘chat sessions, “ask the Expert” facility); iii) peer-to-peer (e.g. peer-to-peer discussions, live-chat). Furthermore, the details recorded about the advisor were study-specific descriptions of their role (e.g. moderator), alongside any prerequisite qualifications or training given.
3. *Supplementary modes*. such as i) email; ii) phone; iii) SMS; or iv) videoconferencing.

### Data analysis

To resolve questions 1 & 2, relevant data on intervention features was grouped together and synthesised using a narrative approach. For question 3, quantitative synthesis using group means and standard deviations were extracted. If this data were not available, study authors were contacted. If required data could not be obtained, that measurement was excluded from the calculations. For studies reporting post-intervention means at multiple time-points, the first post-intervention time-point (e.g. at the conclusion of intervention exposure) was used in calculations. Any studies that reported follow-up data were pooled for separate meta-analysis per outcomes. To address question 4, we contemplated meta-regression using potential modifiers where at least ten studies reported the same outcome. Intervention approach, mode of delivery and participation type (individual vs. group participation) were identified as viable modifiers.

Meta-analysis using post-intervention measurements were conducted when outcome data were reported by at least two RCTs. We calculated the inverse variance method of Hedges g standardised mean differences (SMD) with 95% confidence intervals, using RevMan version 5.4 for effect-size pooling. The fixed effect model was reported If there were less than five studies (k < 5) in the meta-analysis, or the random-effects model for those with five studies or more (k≥5), in line with recommendations.^57^ Heterogeneity was assessed using the I² statistic,^58^ with an I² calculation of ≤25% considered low: ≤50% considered ‘moderate’; and >50% considered high.^132^

### Risk of bias in individual studies

On account of the variation of study-designs, we assessed the quality of included studies using the Integrated quality Criteria for the Review of Multiple Study designs (ICROMS).^59^ Individual assessment items are stipulated within seven dimensions, in which items are either specified for particular study designs (e.g. 2A: allocation adequately controlled for RCT's only) or applicable to all types (e.g. 3E: outcome measures assessed blindly). Relevant items are scored (2  =  criterion met; 1  =  unclear; 0  =  criterion not met), which are then summed to provide a ‘global quality score’ for each paper. These totals were assessed against the specified threshold scores (60% of the total available) for each study design, detailed in a ‘decision matrix’, which also stated individual ‘mandatory criteria’ items for study-types. Threshold scores are 18 out of a possible 30 for CBA's and 22 out of 36 for RCT's or NCBA's.

NB conducted an initial quality assessment. HMB conducted an independent assessment on a 20% random sample of all included studies. Both NB and HMB compared their assessments for this random sample. Any unresolved discrepancies or disagreements were settled by the review team.

### Evidence quality

For studies incorporated into meta-analyses, the Grading of Recommendations, assessment, Development and Evaluation (GRADE) framework was applied to assess the evidence quality.^53^ This approach uses five domains: risk of bias, inconsistency, indirectness, imprecision and publication bias to appraise evidence quality. This is reflected in one of four levels: ‘high’; ‘moderate’; ‘low’ or ‘very low’.

## Results

**Figure 1.**
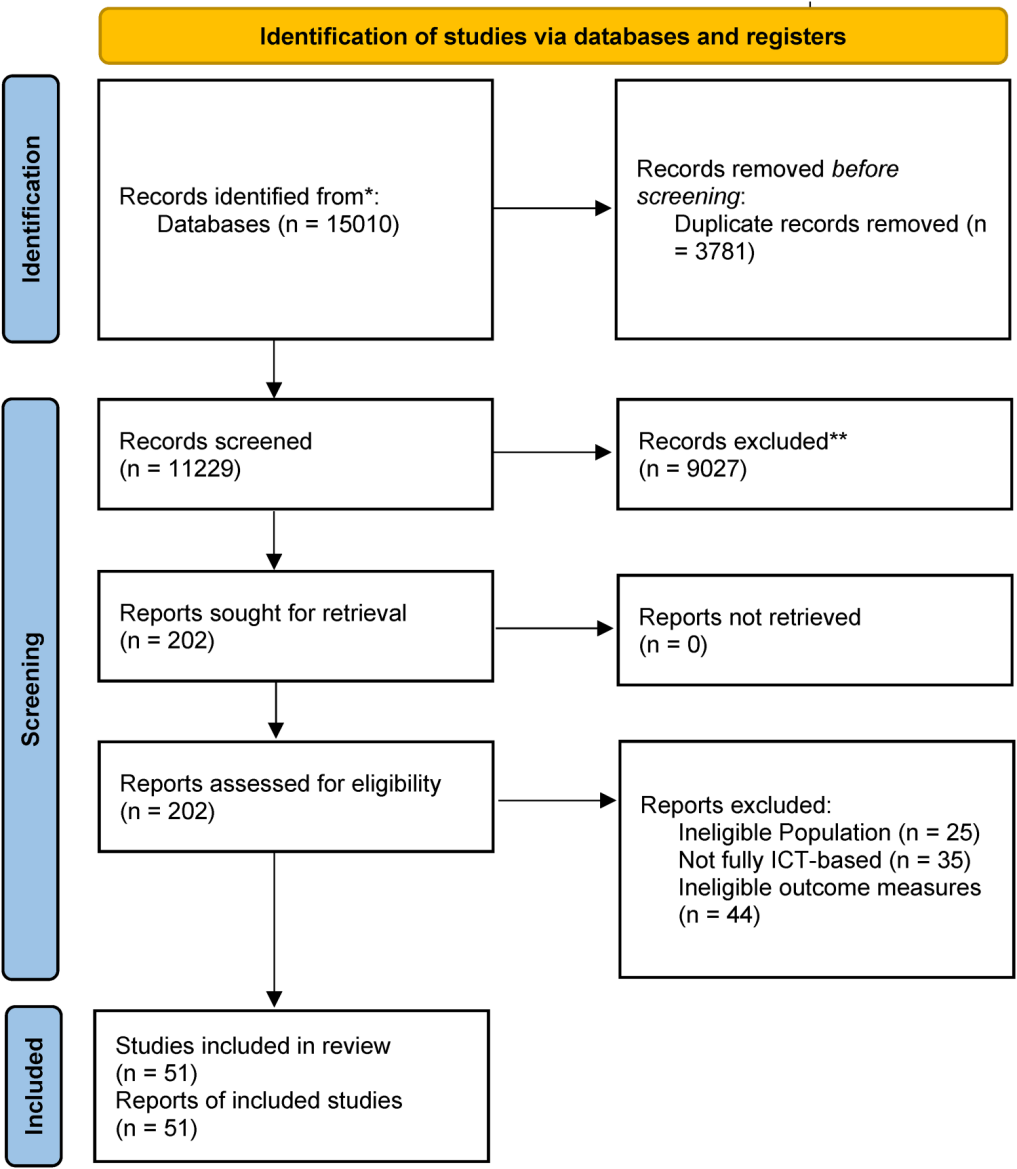
Prisma flowchrt for the identification of studies.

The initial search generated 15,010 results. Following deduplication and screening via abstract and title, 202 references were progressed for full-text screening by the first author (NB). Following calibration with another author's (HMB) independent screening, 51 studies (all unique study datasets) were assessed as fully meeting eligibility criteria. The full screening process is illustrated in Figure 1.

Of the 151 excluded full-text studies, the 40 studies classed as ‘near-misses’ are detailed in Appendix 3, with reasons for their exclusion. The most frequent reasons for exclusion were: >50% of intervention not internet-facilitated (k  =  8), non-caregiver focused intervention or measures (k  =  5) and outcome criteria not met (k  =  8).

### Description of studies

Within the 51 eligible studies, 48 different interventions are reported; three interventions were reported in more than one study, albeit involving different study populations; ‘Tele-STAR’,^83,95^ ‘Caring for Others’^94,95^ and DEM-DISC’.^97,99^ The final list consisted of 26 RCTs, seven controlled before–after (CBA) studies and 18 non-controlled before after (NCBA) studies. Study-duration varied, as did whether authors reported weeks, months or years. The briefest studies lasted less than one month^76,94,101^; the longest was two years in length.^8^ Geographically, almost half the studies (k  =  25) were conducted in North America and another 19 in Europe. Of the remaining studies, three were carried out in South-Asia, one in South America, one in Australia, one in India and one in Iran.

Most studies limited outcome data collection to pre- and post-intervention (k  =  35), with few studies reporting outcomes mid-intervention (k  =  8) or follow-up (k  =  10) ranging from two weeks^83^ to 42 weeks.^66^ We identified 38 individual outcome domains quantified using 92 different outcome-measures. Depression was the most frequently measured (k  =  32) using eight different scales, followed by burden (k  =  26) using six different scales and mastery (k = 13) using six different scales.

An overview of study characteristics, listed by study lead author, is presented in Table 1

**Table 1.** Summary of Included Studies.

**Lead Author Name (Study Ref), Country**	**ND**	**Study Duration & Design**	**Intervention Intensity, Frequency & Length**	**Individual/ Group/ Dyadic Participation**	**Delivery Features**	**Control Arm Type**	**Outcome measure Time-points**
**Austrom** **(60), United States**	Alzheimer's Disease (AD)	6 month NCBA^3^	Weekly support group over 6 months	Group	Web-based video support group accessed using desktop computer equipment	None	Pre & Post (intervention) PHQ-9^4^ GAD-7^5^ SF-36^6^ CBS^7^ RCSES^8^
**Blom** **(61), Netherlands**	Dementia	6 month RCT^1^	Eight lessons with homework followed by booster session one month after, over 6 months	Individual	Multimedia internet course & secure application used for returning caregiver-completed worksheets. All accessed via an Internet-connected computer	Information E-bulletins	Baseline, after fourth lesson, post- intervention: CES-D^9^ HADS^10^ Self-Perceived Pressure from Informal Care scale RMPBC^11^ SSCQ^12^
**Boots** **(62), Netherlands**	Dementia	2 year RCT	Face-to-face sessions delivered at start and of intervention Modules were released every two weeks, although participants could complete at their own pace Over 8 weeks	Individual	Multimedia tailored online thematic modules, email- delivered feedback. Peer access through online forum. No device specified.	Usual care: non-frequent counselling sessions during 8 weeks	Pre & Post- intervention: Main: CSES^13^ CES-D Secondary: PMS^14^ ICECAP-O^15^ HADS
**Chiu** **(63), Canada**	Dementia	6 month NCBA	Participant determined frequency of use over 6 months	Individual	Website provides access to information handbook using internet- connected computer. Email messaging with professional	None	Pre & Post intervention: BSFC^16^ RMPBC CES-D; MSPSS^17^ PAC^18^ CCS^19^
**Cristancho-Lacroix** **(64), France**	Alzheimer's Disease (AD)	6 month RCT	Twelve thematic sessions: sequentially and weekly unblocked once the previous session entirely viewed over 3 months	Individual (psychoeducation) group (social forum)	Multimedia website provides information modules and anonymised peer access via an online forum. Accessed via Internet- connected computer	Usual care; 1xvisit to geriatrician & wait-list receive access at end of intervention	Pre & post intervention, 6-month follow-up: PSS-14^20^ RSCSE RMPBC ZBI^21^ BDI^22^ NHP^23^
**Czaja** **(65), United States**	Alzheimer's Disease (AD)	5 month RCT	Six one-hour monthly sessions; video seminars released monthly over 5 months	Individual (video seminars) Group (peer support sessions)	Video seminars & live videophone support sessions, both accessed via a CISCO IP 7900 videophone	Information: Mailed package of printed Alzheimer's caregiving content	Pre & Post; CES-D 10 RMBPC Risk Appraisal Questionnaire PAC Social support; unspecified ten-item scale
**Dam** **(66), Netherlands**	Alzheimer's Disease & Related Disorders (ADRD)	2 year NCBA	Participant determined frequency of use over 16 weeks	Individual	Online social support platform; social networking function, caregiver- completed information with facility for peer response; multimedia information	None	Baseline, 8 weeks, 16 weeks & 42 weeks follow up: MSPSS SSL^24^ LS^25^ SSCQ
**Dang** **(67), United States**	Dementia	12 month NCBA	Minimum of monthly contact between care co-ordinator and dyad; otherwise participant determined frequency of use; over 12 months	Dyadic	Computer- Telephone Integration System (CTIS) providing access to caregiving information, automated surveys by which caregivers were monitored, alongside facility to make & receive calls	None	Pre & Post: ZBI CES-D COPE^26^ SF-36
**Duggleby** **(68), Canada**	ADRD	6 month RCT	Participant determined frequency of use over 3 months	Individual	Platform provides access to information & areas to enter individual information. Access via computer, tablet or mobile.	Usual care & information: Alzheimer's printed educational booklet	Pre & post, 6 month follow-up: SF12v2^27^ Secondary: GSES^28^ HHI^29^ HSSUI^30^
**Finkel** **(69), United States**	ADRD	Six- month RCT	Two in-home sessions (initial and last session) and 12 sessions conducted via the CTIS system. There were eight individual educational/skill building sessions and six support group sessions (six caregivers and a facilitator), which were interspersed. Over 6 months	Individual (psychoeducation) group (social forum)	Computer- Telephone Integration System (CTIS) providing access to caregiving information, facility to make & receive calls, send & retrieve messages, conference with several people simultaneously	Basic printed educational materials'; two check in phone-calls three & five months post-randomization	Pre & post, 6 month follow-up: CES-D RMPBC Caregiver Health & Health Behaviours scale RSS^31^
**Fowler** **(70), United States**	ADRD	12 month RCT	Weekly posting online of educational information, with a blog monitored daily by the research team over 4 months	Individual	Multimedia website providing access to caregiving information, facility to upload data from sleep actigraphy band & peer- professional support via a blog	Upload data from sleep actigraphy band only	Pre & post: GSES Insomnia Severity Index
**Fowler-Davis** **(71), United kingdom**	Dementia	4 month NCBA	Participant determined frequency of use over 4 months	Individual	Digital plug installed with a 'routinely used' electrical device (e.g. kettle), monitored via mobile application	None	Pre & post: Warwick- Edinburgh Mental Wellbeing Scale (sf) ZBI
**Griffiths** **(72), United States**	Dementia	8 week NCBA	Daily internet- delivered video modules (six per week); weekly group videoconferences over 6 weeks	Individual (psychoeducation) group (social forum)	Online video training modules delivered on iPads	None	Pre & post: ZBI CES-D STA-I^32^ RMPBC PMS
**Gustafson** **(73), United States**	Dementia	6 month RCT	Participant determined frequency of use, although researchers encouraged minimum once per week over 6 months	Individual (psychoeducation) group (social forum)	Website provides access to: online forum; professional messaging service; access to multimedia information & areas to enter individual information, accessed via computer	Information: A printed book for family caregivers of dementia patients	Pre & post: PHQ UCLA LS^33^ GADS MOS Social Support Survey Satisfaction with Decision Scale Lawton et al.'s caregiver appraisal scale.
**Hattink** **(74), Pan-European**	Dementia	10 month RCT	Participants were invited to take part in their national Facebook community (communities were created for all nationalities of users) They were asked to follow at least 4 modules, take part in the end of module knowledge, complete the interactive in-module exercises, & watch a selection of in-module videos. Participants were advised to follow at their own pace/ over 4 months	Individual (psychoeducation) group (social forum)	Platform for interactive e learning and Facebook & LinkedIn-enabled peer-peer/professional communication. No device specified	Waitlist; receive access to intervention following post-test measurements	Pre & post ADQ^34*^ ADKS^35^ SSCQ IRS^36^
**Hicken** **(75), United States**	Dementia	4–6 month CBA^2^	Intervention content accessed 3 days per week for approximately 10-15 minutes over 4–6 months	Individual	Internet-provided multimedia information modules; caregiver health & wellbeing assessments, remotely monitored. Accessed via computer	Telephone calls	Pre & post: ZBI MARWIT^37^ PHQ Conflict / hardship questionnaire DIS^38^
**Kajiyama** **(76), United States**	ADRD	RCT	Participant determined frequency of use; no time limit specified for module completion	Individual	Multimedia e-training program with an emphasis on skills training. Accessed on 'any type of computer'.	Information: Multimedia dementia-information website, without training component	Pre & post: PSS RMPBC CES-D PQOL^39^
**Kajiyama** **(77), United States**	Alzheimer's Disease (AD)	4 week NCBA	Recommended one episode per week: but not monitored; self-paced over 4 weeks	Individual	Online Spanish-language telenovela	None	Pre & post: PSS CES-D Knowledge Scale
**Kales** **(78), United States**	Dementia	2 month RCT	Participant determined frequency of use over 1 month	Individual	Website provides an algorithm-generated 'prescription' using information entered, caregiving information & a daily messaging feature. Accessed via iPad	Waitlist; receive intervention access one month from baseline assessment	Pre & post: NPI-Q^40^ CES-D ZBI Negative Communication Scale Relationship Closeness Scale
**Khazaeili** **(79), Iran**	Multiple Sclerosis (MS)	6 month RCT	8 weekly two-hour sessions over 8 weeks	Group	Webinar; educational & audio content delivered via the Telegram application	No exposure	Pre & post, one month follow up: BAI-II^41^ BDI-II CBI^42^ FFMQ^43^
**Kwok** **(80), Hong Kong**	Dementia	9 week NCBA	8 weekly sessions with completion of online worksheets, alongside direct messaging to the counsellor The counsellors responded to the messages of the participants within 48 hours. There was no upper limit to the total number of messages over 9 weeks	Individual	A website with multimedia information; counselling component delivered using online messaging	None	Pre & Post Chinese versions of: NPI-Q RSCSE: RDB (disturbing behaviour management); CUT (controlling upsetting thoughts)
**Lai** **(81), Hong Kong**	Dementia	7 week RCT	Seven weekly training workshops over 7 weeks '	Group	Website provides access to online group forum	Face-to-face delivery	Pre & post: GHQ^44^-30 Alzheimer's Disease Knowledge test ZBI WHO Quality of Life Measure-brief version
**Laver** **(82), Australia**	Dementia	4 month RCT	8 consultations lasting approximately 60 minutes over 16 weeks	Dyadic	Videoconferencing software accessed via laptop, tablet or smartphone	Face-to-face delivery	Pre & post: CMI^45^ Secondary: PCS^46^ Caregiver Assessment of Function & Upset Caregiver Behavioural Occurrence & Upset Scale Usefulness evaluation
**Lindauer** **(83), United States**	Alzheimer's Disease (AD)	4 month NCBA	8 weekly sessions over 8 weeks	Individual	Online video- conferencing, via computer, laptop or smartphone. Caregiver's also write notes in a workbook to be shown to the screen	None	Pre, halfway, post & 2 month follow up: CES-D ZBI QOL-AD^47^ RMPBC
**Marziali** **(84), Canada**	Dementia	6 month RCT	A 1-hour group therapist- facilitated video-conferencing weekly for 10 weeks. Subsequently, 12 weekly video-conferencing sessions facilitated by group member over 22 weeks	Group	A password-protected Web site with links to (a) disease-specific information, (b) private e-mail, (c) a question-and-answer forum, and (d) a videoconferencing link	No exposure	Pre & post: SF-12 CES-D Stress RMBPC MSPSS
**Marziali** **(85), Canada**	Dementia	6 month CBA	Weekly log on for 1 hour over 20 weeks	Group	Password-protected Web site with link to video-conferencing and online dementia handbook	Online text-based chat group and educational dementia care videos	Pre & post: EPQ-R^48^ RCSES MSPSS HSQ-12^49^ CES-D SMAF^50^
**McKechnie** **(86), United Kingdom**	Dementia	12 week CBA	Participant determined frequency of use over 12 weeks	Individual	Online forum	None	Pre & post: PHQ-9 GAD-7 SQCRC^51^
**Meichsner** **(87), Germany**	Dementia	2 year RCT	One therapist message and one participant reply weekly. Follow-up messages for non-response after two days over 8 weeks/	Individual	Online messaging using a secure internet platform	Waitlist; receive intervention post follow-up assessment	Pre & post, follow-up (5 months post-baseline): CES-D CGS^52^ Psychosocial Resource Utilization Questionnaire for Family CG's of People with Dementia Burden of care and emotional well-being=visual analogue scale
**Metcalfe** **(88), Pan-European**	Young-onset Alzheimer's Disease (AD) or Frontotemporal degeneration (FTD)	12- week RCT	Programme available online 24 hours a day. Usage determined by participant over 6 weeks	Individual	Web-based multimedia information	Waitlist; access given after 6 weeks	Pre & post: PSS RCSES RMPBC BSFC EQ-5D-5L^53^
**Nunez-Naveira** **(89), Pan-European**	Dementia	3 month RCT	Participant determined frequency of use over 3 months	Individual	Online application providing multimedia information, questionnaires for caregivers to complete for personalization and a social network. Accessed via computer, smartphone or tablet	No exposure	Pre & post: CES-D CCS RCSS^54^
**O’Connor** **(90), United States**	Dementia	8 week NCBA	Each group convened weekly for one hour over 8 weeks	Group	Platform providing virtual reality and avatar section. Communication text-only. Accessed via computer	None	Pre & post: UCLA LS GDS^55^ ZBI PSS
**Pagan-Ortiz** **(91), United States**	Dementia	1 month RCT	4 sessions of approximately 1–1.5 hours over 1 month	Individual	Website providing access to multimedia information, comment section for professional & peer-interaction	Printed educational materials on Alzheimer's caregiving	Pre & post: PMS LSNS^56^ ZBI CES-D Knowledge assessment
**Park** **(92), South Korea**	Dementia	3 month CBA	Frequency of use encouraged minimum once per week, although participant determined over 4 weeks	Individual	Information content delivered via mobile application	Information: Printed handbook version of the application's contents	Pre & post, 2 week follow up: Stress via saliva cortisol levels Revised Piper Fatigue Scale Sleep efficiency ZBI NPI
**Schaller** **(93), Germany**	Dementia	18 week NCBA	Minimum once-a-week usage over 12 weeks	Individual	Application provides access to information modules; self-completion unmonitored information tools; professional access via messaging tool	None	Pre & post: BSFC EQ-5D-5L
**Sikder** **(94), United States**	ADRD	4 week NCBA	Participant determined frequency of use, although recommended to listen to audio sessions twice a day for the first week and daily thereafter over 4 weeks	Individual	Mobile application providing access to audio sessions and written content	None	Pre & post, including weekly in-app measures: QIDS^57^ PNAS^58^
**Thomas** **(95), United States**	Dementia	12–18mth NCBA	E-AD: Caregiver completed weekly online surveys over 12–18mths: T-STAR; eight sessions, timeframe not reported	Individual	E-AD: the ORCTECH home-based computing system with data collection from installed sensors wirelessly connected to a monitor less PC T-Star: videoconferencing link	None	Pre & post; E-AD FAQ^59^ NPI ZBI-12; T-STAR: RMPBC CES-D 10 ZBI-4;
**Torkamani** **(96), Pan-European**	Dementia	Six- month RCT	Participant determined frequency of use over 6 months	Individual (psychoeducation) group (social forum)	Platform provides access: multimedia information, peer access via online forum, participant- entered information monitored by clinicians; direct messaging to professionals. Access via laptop	No exposure	Pre, 3 month, post: ZBI; NPI BDI Zung Depression Self Rating Scale EQ5D QOLS^60^ platform assessment
**van der Roest** **(97), Netherlands**	Dementia	6 month CBA	Participant determined frequency of use over 2 months	Individual	Web-based interactive search-engine based social chart providing general, local & tailored information. Accessed via computer	No exposure	Pre & post.: GHQ-28 CES-D SSCQ PMS Knowledge questionnaire Evaluation assessment
**van Knippenberg** **(98), Netherlands**	ADRD	4 month RCT	ESM self-monitoring at random times for 3 days per week & received standardized ESM-derived feedback on personalized patterns of positive affect every 2 weeks during a face-to-face session with a coach. over 6 weeks	Individual	ESM: Participants responded to palmtop-generated alerts to complete questionnaires, also completed on the palmtop. Face-to-face feedback following two weeks of monitoring	Pseudo intervention: ESM monitoring without feedback Control: usual care of low-frequent counselling sessions	Pre & Post: SSCQ PMS RMPBC DRS^61^
**van Mierlo** **(99), Netherlands**	Dementia	12 month RCT	Participant determined frequency of use over 12 month s	Individual	Web-based interactive search-engine based social chart providing information based on information entered, links to relevant organizations,; content posted by participants monitored by professionals	No exposure; received advice from case managers who also did not have access	Pre & post: SSCQ CES-D PMS University of South Carolina Longitudinal Study of Three-Generation Families measures of positive affect HADS-7 The 12-item neuroticism domain of the NEO Five-Factor Inventory; emotional instability 44-item UCL^62^
**huis in het Veld** **(100), Netherlands**	Dementia	12 week RCT	Family caregivers received 3 personal email contacts with a specialist dementia nurse. Were also sent links to six online videos and six e-bulletins, over 12 weeks	Individual	Professional contact, video links and e-bulletins all sent via email	Medium intervention: online videos & e-bulletins Control: information e-bulletins only	Baseline/6 weeks/12 weeks: SSCQ
**Wijma** **(101), Netherlands**	Dementia	4 week NCBA	13 minute VR simulation experienced at a local healthcare organisation, then 3×20 min e-courses completed at home within three weeks; total of four weeks	Individual	Virtual reality (VR) movie accessed through a VR-device and e-course for which no device stipulated	None	Baseline/6 weeks/12 weeks ADQ Secondary TOA^63^ DRS
**Wilkerson** **(102), United States**	Alzheimer's Disease (AD)	3 month CBA	Participant determined frequency of use, weekly participation encouraged over 6 weeks	Group	Web-based application to join a Facebook social network. Separate email reminders	Online interaction with researchers only	Pre & post: ZBI-12 PSS-14 RSCSE MOS^64^
**Zimmerman** **(103), United States**	ADRD	6 month CBA	Participant determined frequency of use over 6 months	Individual	Website providing multimedia information, separate reminders sent by email	A printed book with the same, but more concise, content	Pre, 3 months, post CCSM^65^ ZBI-12 PHQ-9 GAD-7
**Moskowitz** **(104), United States**	Dementia	12 month RCT	Weekly sessions over 6 weeks	Individual	Live online webinar accessed via tablet	Waitlist; completed assessments during waiting time. Access after 6 weeks	Pre, three months, post: DES ZBI-22; GHS^66^ Neuro-QOL^67^ PSS
**Baruah** **(105), India**	Dementia	3 month RCT	Participant determined frequency of use, although encouraged to complete at least five lessons, over 3 months	Individual	Multimedia e-learning, where caregivers complete exercises, for which they received instant feedback	Information: Education only e-book based on an Alzheimer's Disease International/WHO brochure	Pre & post: ZBI-12 RIS Eldercare Self-efficacy Scale Mastery Scale CES-D 10 ADQ
**De Wit** **(106), Netherlands**	Amyotrophic Lateral Sclerosis	6 month RCT	6×1.5hrs modules could be completed within 1–2 weeks, over 3 months	Individual	Fixed sequence of online modules where caregivers complete exercises, for which they received feedback. Peer contact through private messaging and a forum	Usual Care	Pre, post, 6 months HADS ZBI-12 CarerQoL^68^ RCSES
**Brunisma** **(107), Netherlands**	Young-onset Alzheimer's Disease (AD)	NCBA	Caregivers follow each module whilst choosing 4 thematic modules, over 8–10 weeks (this is flexible)	Individual	Multimedia tailored online thematic modules, email- delivered feedback. Peer access through online forum. No device specified.	None	Pre, post HADS PSS GSES PMS
**Halstead** **(108), USA**	Multiple Sclerosis	3 month NCBA	6×45 minute weekly sessions, over six weeks	First & last modules: dyadic, Remaining four: individual	Secure, web-based portal MS Hub, through which caregivers accessed related program software e.g. videoconferencing	None	Pre, post, 3 months Connor Davidson Resilience Scale General Life Satisfaction Scale PNAS Burns Relationship Satisfaction Scale HADS PSS MSSS^69^ SCQ ZBI-22
**Han** **(109), USA**	Dementia	3 month NCBA	Weekly one-hour sessions over 3 months	Individual	Sessions delivered via Zoom video-conferencing using a computer or smartphone	None	Pre, post, DASS-21^70^ ZBI-12 EMAS^71^ EACQ^72^ AAQ-II^73^ CFQ-7^74^
**Romero-Mas** **(110), Spain**	Alzheimer's Disease (AD)	10 month NCBA	Participant determined frequency of use over 10 months	Group	All content and activities accessed using a mobile application	None	Pre, post WHOQoL-BREF^75^

Randomised Controlled Trial (RCT)^1^; Controlled Before-After (CBA)2; Non controlled-Before-After (NCBA)^3^; Patient Health Questionnaire (PHQ-9)^4^; General Anxiety Disorder (GAD-7)^5^; Short Form-36 (SF-36)^6^; Caregiver Burden Scale (CBS)^7^; Revised Caregiver Self-Efficacy Scale (RCSES)^8^; Center for Epidemiologic Studies Depression scale (CES-D)^9^; Hospital Anxiety and Depression Scale (HADS-A)^10^; Revised Memory and Behavior Problems Checklist (RMPBC)^11^; Short Sense of Competence Questionnaire (SSCQ)^12^; Caregiver Self-Efficacy Scale (CSES)^13^; Pearlin Mastery Scale(PMS)^14^; Investigation Choice Experiments for the Preferences of Older People (ICECAP-O)^15^; Burden Scale for Family Caregivers (BSFC)^16^; Multidimensional Scale of Perceived Social Support (MSPSS)^17^; Positive Aspects of Caregiver (PAC)^18^; Caregiver Competence Scale (CCS)^19^; Perceived Stress Scale (PSS-14)^20^; Zarit Burden Interview (ZBI)^21^; Beck Depression Inventory (BDI)^22^; Nottingham Health Profile (NHP)^23^; Social Support List (SSL)^24^; Loneliness Scale (LS)^25^; Brief Cope (COPE)^26^; Short Form-12 item [version 2] health survey^27^; General Self-Efficacy Scale (GSES)^28^; Herth Hope Index (HHI)^29^; Health & Social Services Utilization (HSSUI)^30^; Received Social Support scale (RSS)^31^; State-Trait Anxiety Inventory (STA-I)^32^; (UCLA LS)^33^; Approaches to Dementia Questionnaire (ADQ)^34^; Alzheimer's Disease Knowledge scale (ADKS)^35^; Interpersonal Reactivity Scale (IRS)^36^; Marwit-Meuser Caregiver Grief Inventory-Short Form (MARWIT)^37^; Desire to Institutionalize Scale (DIS)^38^; Perceived Quality of Life Scale (PQOL)^39^; Neuropsychiatric Inventory (NPI-Q)^40^; Beck Anxiety Inventory (BAI-II)^41^; Caregiver Burden Inventory (CBI)^42;^ Five Facet Mindfulness Questionnaire (FFMQ)^43^; General Health Questionnaire 30 (GHQ-30)^44^; Caregiver Mastery Index (CMI)^45^; Perceived change Scale (PCS)^46^; Quality of Life in Alzheimer's Disease (QOL-AD)^47^; Eysenck Personality Questionnaire Revised (EPQ-R)^48^;Health status questionnaire (HSQ-12)^49^; Functional Autonomy Measurement System (SMAF)^50^; Scale for the Quality of the Current Relationship in Caregiving (SQCRC)^51^; Caregiver Grief Scale (CGS)^52^; EuroQoL (EQ-5D-5L)^53^; Revised Caregiving Satisfaction Scale (RCSS)^54^; Geriatric Depression scale (GDS)^55^; Lubben Social Network Scale (LSNS)^56^; Quick inventory of Depressive Symptoms (QIDS)^57^; Positive & Negative Affect scale (PNAS)^58^; Functional Activities Questionnaire (FAQ)^59^; Quality of Life Scale (QOLS)^60^; Dyadic Relationship Scale (DRS)^61^; Utrecht Coping List (UCL)^62^; Trust in our Own Abilities (TOA)^63^; Medical Outcomes Survey (MOS)^64^; Caregiver Confidence in Symptom Management (CCSM)^65^; Global Health Scale (GHS)^66^; The Quality of Life in Neurological Disorders (NeuroQOL)^67^; Care- Related Quality of Life (CarerQoL)^68^; Modified Social Support Survey (MSSS)^69^; Depression, Anxiety and Stress Scale (DASS)^70^; Engagement in Meaningful Activities Survey (EMAS)^71^; Experiential Avoidance in Caregiving Questionnaire (EACQ)^72^; Acceptance & Action Questionnaire II (ACQ-II)^73^; Cognitive Fusion Questionnaire (CFQ-7)^74^; (WHOQoL-BREF)^75^

### Population characteristics

A total of 3180 informal caregivers were enrolled across all studies. For RCTs, or CBAs that incorporated comparative groups, the mean number of participants was 43 (SD = 36.72) in intervention and 41 (SD  =  27.05) in control arms. In NCBAs, the mean sample size was 28 (SD  =  16.99). All but five studies^63,79,81,83,91^ provided information on gender of participants; female caregiver participants constituted 32%^108^ to 100%^60,90^ of the sample size.

Reporting of other demographic variables such as age, ethnicity or caregiver-patient relationship varied substantially across studies. Where studies reported mean ages, this ranged from 52.9 years (SD  =  11.4) to 72.1 years (SD  =  8.4). For the studies that reported age ranges, the lowest reported was 20, the highest 87. Five studies completely omitted this information. In terms of caregiver's relationship to care recipient, most studies involved spouses or adult-children.

In respect of other socio-demographics (e.g. educational-attainment, monthly income), our ability to synthesize was constrained by the extent of inconsistent reporting. This was also true for other variables of interest, including pre-intervention ownership, familiarity or confidence with internet-facilitated technology. Where the latter was captured, this was principally via qualitative methods, with little quantitative assessment.

Apart from two studies^79,106^ detailing Amyotrophic Lateral Sclerosis (AMS) and one study^108^ detailing MS, all other studies (k  =  48) concerned patients whose diagnosis was either described as

Dementia, AD or Alzheimer's Disease and Related Disorders (ADRD). There were no studies involving HD or PD.

### Interventions: key features and classifications

Intervention duration varied, from interventions lasting four weeks,^77,92,94,101^ to the longest at 12 months^67,99^ in length. The mode intervention duration was six months, although more than half the interventions lasted for three months or less. With reference to delivery-intensity, sessions were most frequently delivered weekly (k  =  19). A further 10 stipulated or recommended minimum usage; either a number of uses or modules to be completed, whilst 15 observed spontaneous usage of the intervention as a measure.

There were 33 interventions in which caregivers’ participated as individuals; eight interventions involved group-participation, whilst four were targeted at caregiving dyads. There were six studies in which some parts were undertaken individually with others undertaken in a group. Where studies employed a comparator group (k = 33), the majority received some form of information only (k  =  13), whilst being assigned to the waitlist group (k  =  6) or simply receiving no intervention (k  =  6) were the other most frequent forms of comparison.

### Approach

Our full categorisation of approach using Chi & Demiris’^54^ classification system is presented in Table 2.

**Table 2.** Categorization of approaches and modes of delivery.

Study No.	Study Design	APPROACH (Chi & Demiris, 2015)						MODE OF DELIVERY (Webb et al., 2010)											
		Approach: Education	Approach: Consultation	Approach: Social	Approach: CBT	Approach: Data Collection	Approach: Clinical Care Delivery	Automated: Enrich	Automated: Tailor	Automated: Follow-Up	Communicative: Scheduled	Communicative: Advisor	Communicative: Peer	Advisor Role	Advisor pre-intervention experience/ qualifications	Supplementary: E mail	Supplementary: Phone	Supplementary: SMS	Supplementary: Videoconferencing
3	RCT	x			x			x	x	x	x			Coach	Principal Investigator	x			
4	RCT	x		x				x			x		x	Facilitator	Psychologist	x			
6	RCT	x		x				x					x			x			
7	RCT	x		x				x			x		x	Educator	Certified interventionist				
10	RCT	x						x											
11	RCT	x		x				x			x		x	Facilitator	Social Worker				
12	RCT	x				x		x				x	x	Moderator	Miscellaneous clinical professionals				
15	RCT	x		x				x	x	x		x	x	Expert	Alzheimer's Information Specialist				
16	RCT	x		x				x	x			x	x	Expert	Dementia Care Professionals				
18	RCT	x						x											
20	RCT	x						x		x	x			Counsellor	Psychologist		x		
21	RCT				x						x			Therapist	No information				
23	RCT	x									x			Facilitator	No information				
24	RCT	x	x								x			Therapist	Occupational Therapist				x
26	RCT	x		x							x		x	Facilitator	Social Worker; Nurse				x
29	RCT				x				x		x			Therapist	Clinical Psychologist	x	x		
30	RCT	x						x									x		
31	RCT	x		x				x					x						
33	RCT	x		x				x				x	x	Expert	No information	x			
38	RCT	x		x		x		x	x			x	x	Expert	Clinical Team				
40	RCT		x			x			x	x		x		Coach	No information				
																			
41	RCT	x				x		x				x		Moderator	Clinical Case Manager				
42	RCT	x				x		x	x	x	x	x		Expert	Nurse	x			
46	RCT				x				x		x	x		Therapist	Certified interventionist				x
47	RCT	x			x			x								x			
48	RCT	x		x				x	x		x	x	x	Coach	Psychologist		x		
2	NCBA	x		x								x	x	Facilitator	Principal Investigator		x		
5	NCBA	x			x			x			x			Therapist	Clinician	x			
8	NCBA	x		x				x					x				x		
9	NCBA	x				x		x		x		x		Expert	Nurse				
13	NCBA					x				x									
14	NCBA	x			x			x			x		x	Facilitator	No information		x		
19	NCBA	x						x						Expert	Miscellaneous clinical professionals				
22	NCBA				x			x	x		x	x		Therapist	Miscellaneous clinical professionals				
25	NCBA	x		x	x					x	x		x	Educator	Dementia Nurse				x
32	NCBA													Facilitator	Psychologist				
35	NCBA	x				x		x				x		Expert	Miscellaneous clinical professionals	x	x		
36	NCBA				x			x											
37	NCBA				x	x		x			x			Educator	Nurse				
43	NCBA	x						x						Educator	Dementia Nurse	x			
49	NCBA	x						x	x		x			Coach	Psychologist; Clinical Case Manager				
50	NCBA	x						x			x			Coach	Social Worker				
51	NCBA				x					x	x	x		Coach	Licensed Counsellor				x
52	NCBA			x				x					x	Moderator	Miscellaneous clinical professionals; experienced caregiver				x
17	CBA	x				x		x	x			x		Care Manager	Miscellaneous clinical professionals				
27	CBA			x				x			x		x	Facilitator	Social Worker; Nurse				
28	CBA			x								x				x			
34	CBA	x						x			x			Moderator	Principal Investigator		x		
39	CBA	x						x									x		
44	CBA			x									x	Moderator	Research Team	x			
45	CBA	x						x		x						x			

We observed that just under half used only one type of approach (k  =  27), with 21 studies using two and two studies^83,96^ employing three approaches and one^95^ using four. Education was the most frequently adopted approach both overall (k  =  38) and in single-approach studies (50%) within which a wide variety of implementation methods was observed. Almost half of the studies made information available without expectation as to what participants consumed or accessed (k  =  17).

Other strategies include modules with homework to be evaluated (k  =  6), the release of newer material following successful completion of modules (k  =  2) or presenting users with more personalized information dependent on data-entered or questions posed (k  =  5). Where studies detailed a combination of approaches the most frequently observed was social (i.e. peer-to-peer) and education (k  =  12).

### Modes of delivery

Our full summary of application of Webb et al.'s^56^ mode of delivery framework is presented in Table 2. Thirty-seven interventions were classified as using a combination of at least one automated and one communication function, whereas 14 only used one of either. The most frequently observed automated function used was the ‘automated enriched’ environment (k  =  38).

In terms of communicative functions, 22 interventions afforded scheduled access to an advisor, who performed a variety of roles such as delivering counselling or therapy (k  =  9), or facilitating timetabled group videoconferencing sessions (k  =  6). Another 17 involved communication with an advisor, albeit the amount or regularity of correspondence was not always stipulated. These roles included providing expert guidance (k  =  8), facilitating content (k  =  8), or delivering therapy or counselling (k  =  7). Where an advisor's prior experience or qualifications were described, the most common were psychologists (k  =  11), nurses (k  =  8) and therapists (k  =  5).

Eighteen interventions incorporated web-based forums to facilitate access to peers (‘peer-peer’ mode). Regarding how interventions were supported by supplementary modes of communication; email (k  =  11), videoconferencing (k  =  5) and telephone (k  =  9) were used.

No single combination of automative & communicative functions predominated, with the most frequent unique classification pairing (k  =  14) being an ‘enriched’ environment accompanied by ‘scheduled’ contact with an advisor. A few interventions also enabled peer access (k  =  4). Within interventions that combined enriched environment with peer access (k  =  15), five also involved ‘scheduled’ contact with an advisor; another five included unscheduled contact.

### Use of theory

We observed a lack of reference to the theoretical-base in underpinning intervention design and implementation, identifying just 14 interventions that specified at least one theory or construct. These are narratively summarised here using the TCS’ classifications. All of the 14 were classified as either referencing theory, such as social learning^62^ and social cognition theory,^70,72^ or using it as a basis for specific aspects (items 1–6). A theory or model of behaviour (e.g. cognitive behaviour theory, 80) was mentioned in four interventions^62,70,80,105^ whilst seven used a theory or predictor (e.g. Meleis’ theory of transition) to select or develop intervention technique.^64,68,72,74,78,92,104^ A theory or predictor (e.g. stress-coping and adaptation paradigms) was used to tailor techniques to recipients in two interventions.^84,85^ From items linking theories or constructs to interventions,^7–11^ at least one theory-relevant construct was linked to the technique in four interventions^64,68,74,78^ and at least one technique was linked to a theory in five interventions.^70,72,80,84,85^ Examples of the theories or predictors extracted include Meleis’ theory of transition,^68^ the DICE (Describe, Investigate, Create, Evaluate) approach^78^; communities of practice model^110^ and Kales’^111^ theoretical framework for reasons and management of BPSD^93^; alongside stress, coping and adaptation paradigms^62,64,72,74,84,85,106^; Social Learning Theory^62^ and Social Cognition Theory.^70,72^

### Risk of bias analysis

As illustrated in Table 3, 32 studies (63%) were scored as meeting the ICROMS criteria, whereas 19 studies (37%) did not score sufficiently. Of the 26 RCT studies, scores ranged from 11 to 31 (mean 23.15, median 24); nine (38%) did not meet the minimum score threshold of 22. Within the six CBA studies scores ranged from 19 to 22 (mean 20.33, median 20), all of which met the minimum score threshold of 18. Furthermore, there were 19 NCBA studies, whose scores ranged from 10 to 23 (mean 20.12, median 21); 11 studies (58%) met the minimum score threshold of 22. Specific sections that consistently received low or no scores were sampling and outcomes. Within these, the criteria not met were lack of allocation concealment, blinding and blinded-assessment of outcome measures. Other criteria lacking evidence were results free of other bias, follow-up of patients and incomplete outcome data addressed. The latter was reflected in the fact that only 26 studies reported attrition rates; ranging from just one participant not completing outcome measures^60,83^ to 67% with incomplete or missing data.^105^ Reasons for dropout and comparative statistics for completers and non-completers were seldom provided.

**Table 3.** Risk of Bias Assessments.

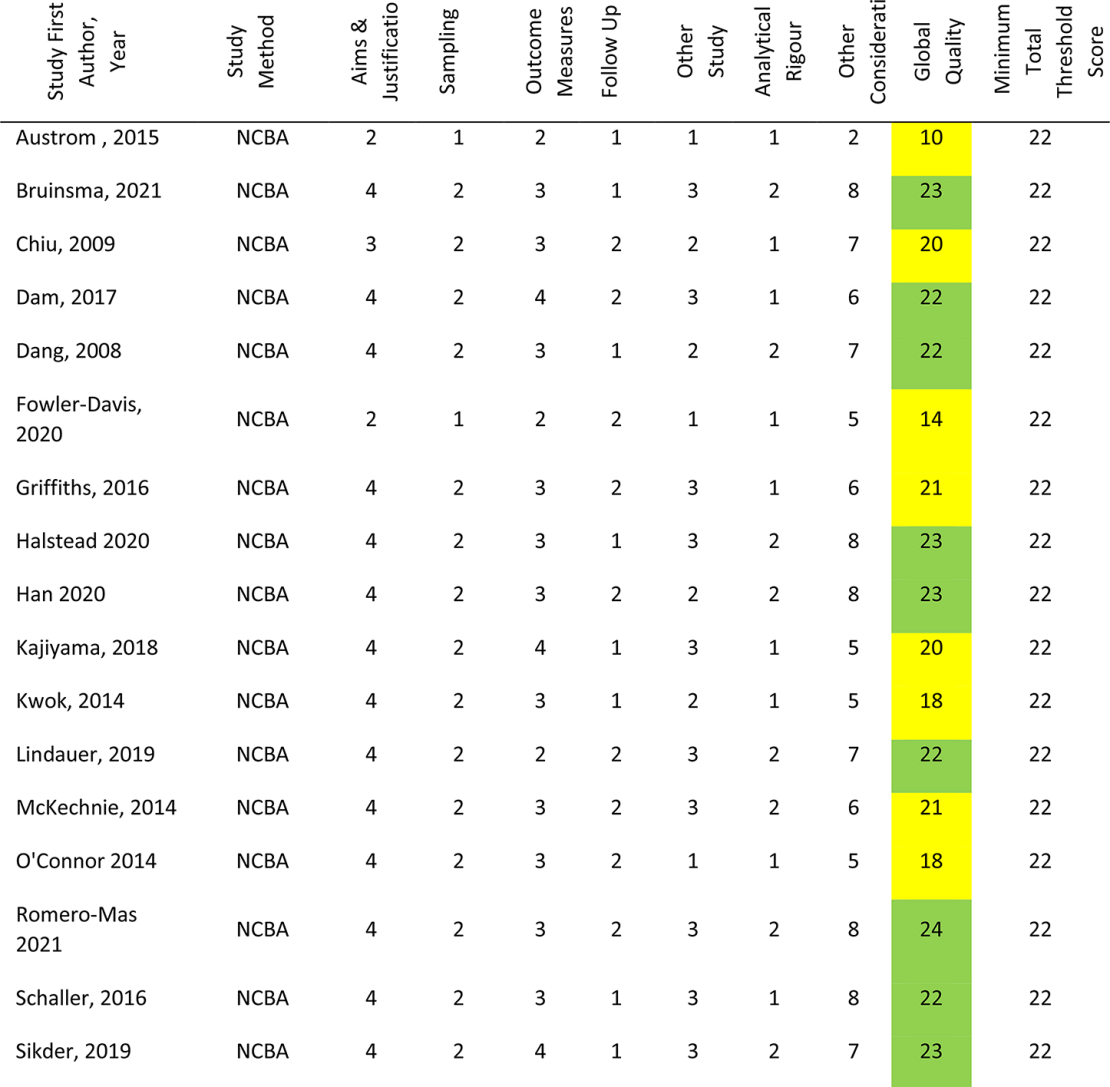
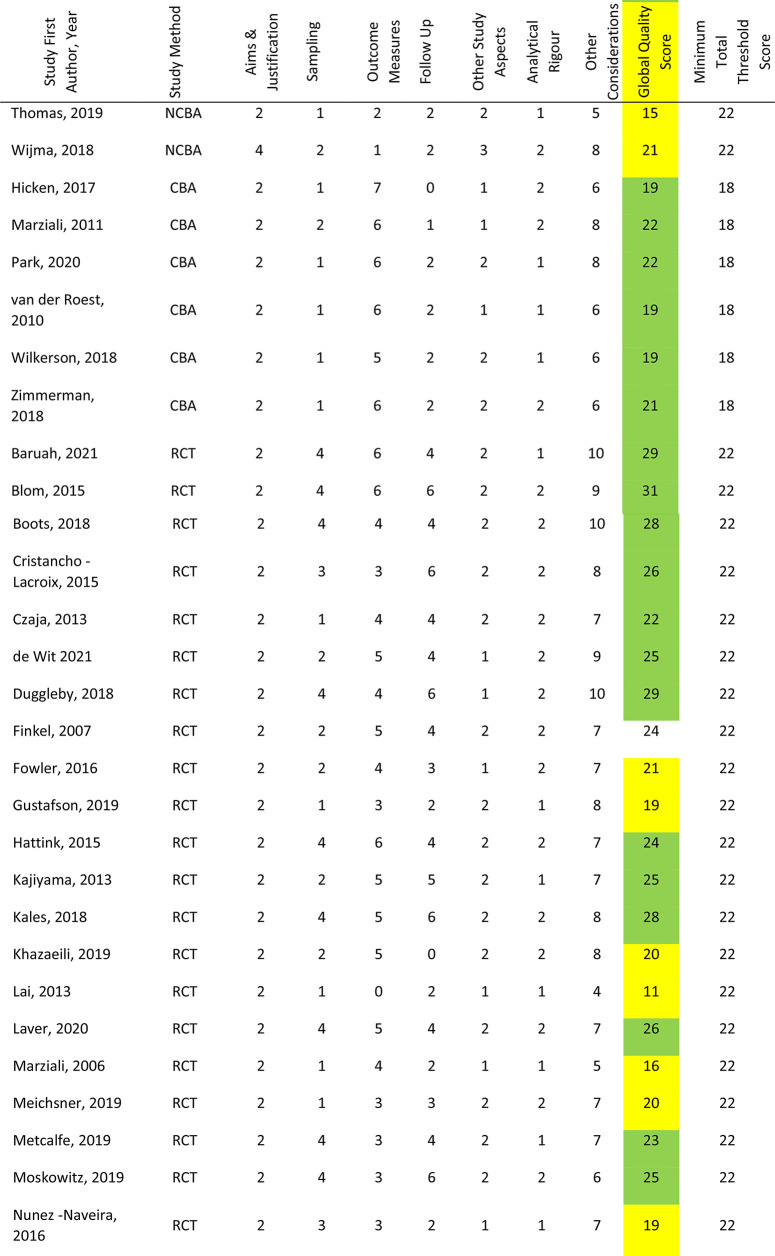
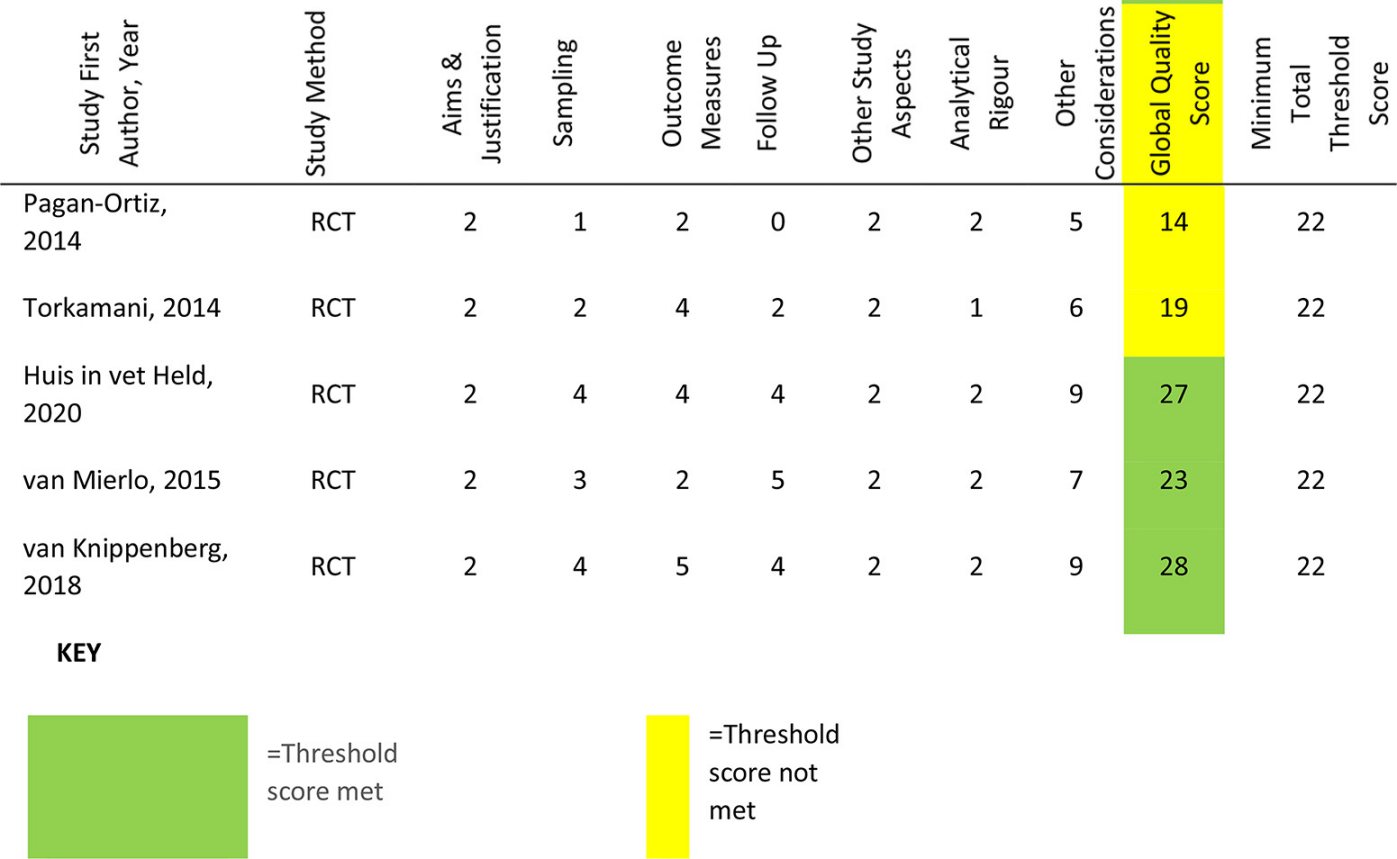

Correspondingly, power calculations to determine minimum number of participants were detailed in just 15 studies; all but one reported achieving their original participant target.

### Effectiveness of internet-facilitated interventions on caregivers’ outcomes

#### Anxiety

**Figure 2.**
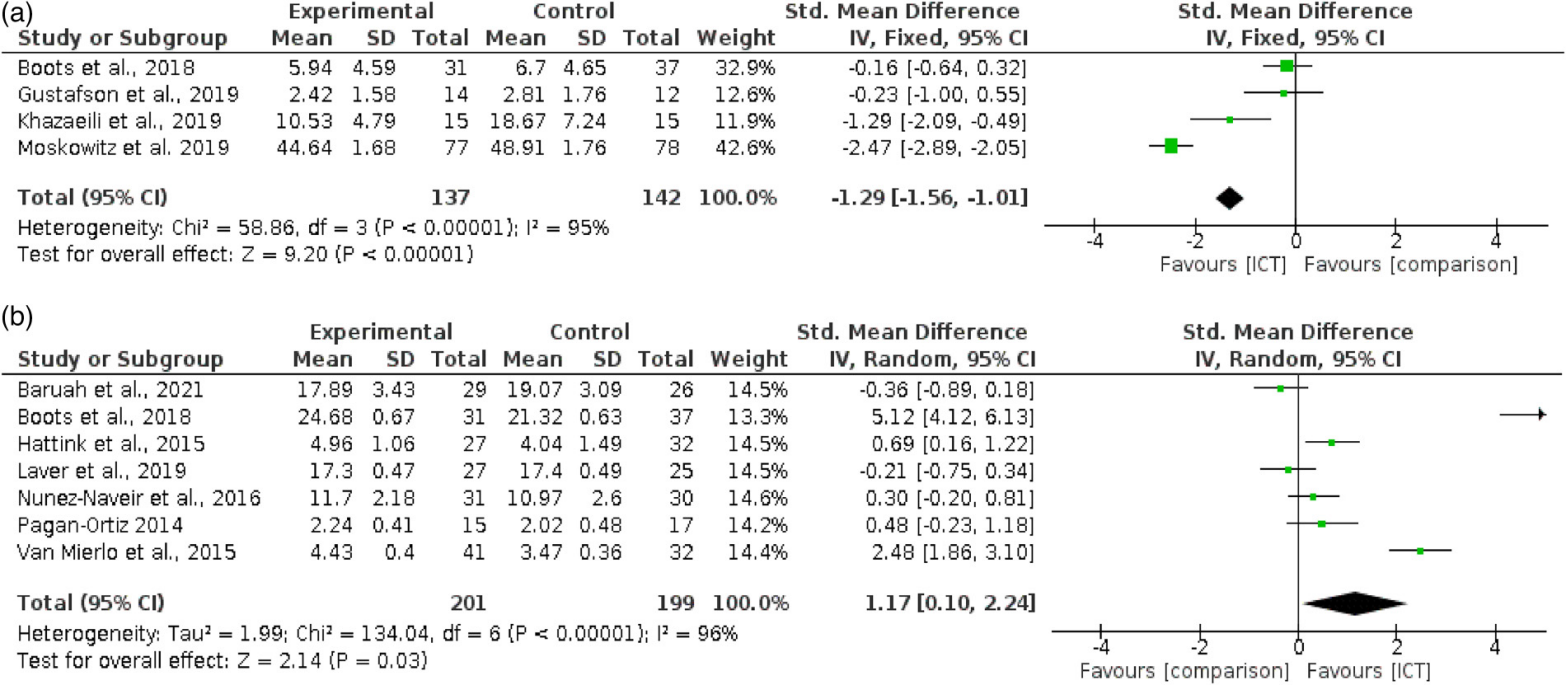
Meta-analysis on caregivers’ psychological outcomes (a) Comparing efficacy of internet-facilitated interventions with comparators on improving caregivers’ anxiety outcomes. (b) Comparing efficacy of Internet-facilitated interventions with comparators on improving caregivers’ sense of mastery outcomes.

As depicted in figure 2 a), 4 studies^62,73,79,104^ reported anxiety outcomes. Comparator groups were either no exposure/information only^62,73,79^ or waitlist.^104^ The fixed-effect meta-analysis demonstrated a significant effect of interventions, compared with controls, on reducing anxiety; (4 RCTs; n  =  279; SMD −1.29, 95% CI; −1.56 to −1.01; P < 0.01; I^2^  =  95%; GRADE quality of evidence very low).

The substantial heterogeneity rate reflects the diverse intervention features within these studies. Intervention length in the American studies was either six weeks,^104^ or 3 months.^73^ In both the European^62^ and Iranian^79^ studies, intervention duration was eight weeks. Two were CBT/psychotherapy-based,^79,104^ whereas one combined psychoeducation with social support^73^ with the other combining psychoeducation with peer support and access to an advisor.^62^ Participants completed two at their own pace^73^ whereas one gave guidance^62^ and one involved weekly scheduled contact with an advisor.^79,104^ In addition, three of the four studies were identified as theory-based^62,73,104^ and the other was an online-adaptation of a face-to-face technique.^79^ We could not fully compare populations due to one study^79^ only providing an age range for the overall sample and no gender-split information.

#### Sense of mastery

Seven RCTs^62,74,82,89,91,99,105^ reported sense of mastery outcome data. Comparator groups were either no exposure/information only,^62,89,91,99,105^ waitlist^74^ or face-to-face delivery.^82^ The random-effects meta-analysis demonstrated a significant effect of internet-facilitated interventions compared to controls, on increasing sense of mastery (7 RCTs; n  =  400; SMD 1.17, 95% CI; 0.1 to 2.24; *P*  =  0.03; I^2^  =  96%; GRADE quality of evidence very low). This is depicted in Figure 2 b).

Two outliers were identified^62,105^ whose CI's did not overlap with the model's overall CI. Without these studies, heterogeneity remained moderate (I^2^  =  61%) reflecting the diverse features across interventions. Of the four European studies,^62,74,89,99^ one collected data to provide personalised information,^99^ two combined psychoeducation with peer support,^74,89^ whilst the remaining combined psychoeducation with peer support and access to an advisor.

These latter delivery modes were also used in the American study.^91^ In the study conducted in India,^105^ caregivers completed interactive educational exercises, whereas the study conducted in Australia^82^ provided consultation to the caregiving-dyad through scheduled bi-weekly appointments. Participants completed four interventions at their own pace^89,91,99,105^ whereas two gave guidance or recommended minimum usage.^62,74^ All were of differing length, from one month^91^ to 12 months.^99^ Whilst all care-recipients were dementia-diagnosed, we were unable to compare populations further due to lack of data on age^89^ and gender split.^91^

### Other outcomes

Seven studies reported burden outcomes. The random-effects meta-analysis demonstrated that there was no significant effect of interventions, compared with controls, on reduction of burden (7 RCTs; n  =  617; SMD −0.47, 95% CI −1.13 to 0.18 *P*  =  0.16; I^2^  =  93%; GRADE quality of evidence very low). Two outlier studies^79,104^ were identified whose CI's did not overlap with the model's overall CI. Without these heterogeneity reduced to low (I^2^  =  9%), although the model remained non-significant (5 RCTs; n  =  432; SMD −0.04, 95% CI −0.16 to 0.25; *P*  =  0.66).

Ten studies reported depression outcomes. The random-effects meta-analysis demonstrated that there was no significant difference between groups at post-intervention measurement (10 RCTs, n  =  656; SMD −0.28, 95% CI −0.81 to 0.26; *P*  =  0.12; I^2^  =  90%; GRADE quality of evidence very low). We identified one outlier study^104^ whose CI's did not overlap with the model's overall CI. Without these heterogeneity reduced to low (I^2^  =  9%), although the model remained non-significant (9 RCTs; n  =  501; SMD −0.05; 95% CI −0.23 to 0.13; *P*  =  0.59).

Five studies reported stress outcomes. The random-effects meta-analysis demonstrated no significant effect of interventions, compared with controls, on reducing stress measurements (5 RCTs; n  =  302; SMD −1.42, 95% CI −2.95 to 0.1; *P* = 0.07; I^2^  =  97%; GRADE quality of evidence very low). Two outlier studies^62,104^ were identified whose CI's did not overlap with the model's overall CI. Without these heterogeneity reduced to low (I^2^  =  0%), although the model remained non-significant (3 RCTs; n  =  179; SMD 0.06, 95% CI −0.23 to 0.35; *P*  =  0.69).

Six studies reported strain outcomes. The random-effects meta-analysis demonstrated no significant effect of interventions, compared with controls, on strain measurements (6 RCT's; n  =  563; SMD −0.07, 95% CI −0.49 to 0.31; *P* = 0.7; I^2^  =  87%; GRADE quality of evidence very low). We did not identify any outlier studies.

Four studies reported HRQoL outcomes using three different measurement instruments. The fixed-effect meta-analysis demonstrated no significant effect of interventions, compared with controls, on increasing HRQoL measurements (4 RCTs; n  =  497; SMD 0.07, 95%CI −0.10 to 0.25; *P*  =  0.4; I^2^  =  0%; GRADE quality of evidence very low).

Four studies reported PQoL measures using four different measurement instruments. The fixed-effect meta-analysis demonstrated that there was no significant effect of interventions on increasing PQoL measures (4 RCT's; n  =  472; SMD 0.01, 95%CI −0.19 to 0.17; *P*  =  0.91; I^2^  =  0%; GRADE quality of evidence very low).

Five studies reported self-efficacy outcomes. The random-effect meta-analysis demonstrated that there was no significant effect of interventions on increasing self-efficacy compared with controls (5 RCTs; n  =  444; SMD 0.92, 95% CI; −0.06 to 1.9; *P*  =  0.06; I^2^  =  95%; GRADE quality of evidence very low). We identified one outlier study^62^ whose CI's did not overlap with the model's overall CI. Without this, heterogeneity reduced to low (I^2^  =  0%), although the model remained non-significant (4 RCTs; n  =  376; SMD 0.13, 95% CI −0.07 to 0.33; *P*  =  0.22).

### Follow-up measures

Usable follow-up data was only reported in three RCTs^64,67,106^ with measurements at six months from baseline. The fixed-effect meta-analyses demonstrated no significant effect of interventions, compared with controls, on either decreasing burden measures (2 RCT's; n  =  155; SMD 0.09, 95%CI −0.23 to 0.41; *P*  =  0.59; I^2^  =  26%; GRADE quality of evidence very low) or increasing self-efficacy measures (3 RCT's; n  =  321; SMD 0.05, 95%CI −0.17 to 0.27; *P*  =  0.67; I^2^  =  0%; GRADE quality of evidence very low).

#### Most efficacious intervention approach and design feature

Despite adequate numbers of studies available for depression outcomes (k  =  10), intended exploratory meta-regression to explore effects of different intervention features were precluded by a lack of appropriately populated groups. Neither intervention approach (e.g. education used k  =  7, not used k  =  3), mode of delivery (e.g. communication with an advisor used k  =  2, not used k  =  8), nor participation type (individual participation k  =  8; group participation k  =  2) yielded the group distributions necessary.

## Discussion

### Principal findings

To our knowledge, this is the first review to include diagnoses of rarer NDs (e.g. HD, MS, ALS) when examining the common features and effectiveness of internet-facilitated interventions aimed at informal caregivers. Yet even though our highly sensitive search retrieved 51 quantitative studies, all but three^79,106,108^ involved ADRD; two involved MS, another ALS. This reflects the lack of research into non-ADRD caregivers, despite the growth of diagnoses for disorders such as PD. Apart from the consistency of reporting the patient's diagnosis, details of other demographic variables were too divergent across studies to be able to derive salient information about those caregivers participating.

Most interventions took place in either North America or Europe (84%). Yet geographical location was an isolated commonality, for the narrative review highlighted an array of approaches, modes of delivery and theoretical bases. Although the majority used an educational approach (75%), an enriched environment (75%) and either scheduled or unscheduled contact with an advisor (76%), between-study differences were apparent in the multifarious ways in which individual features were combined. This incongruity was most visible when quantitatively synthesizing the 20 RCT's that provided usable data. High heterogeneity for both anxiety and mastery outcome meta-analyses reflected disparities such as interventions’ lengths, dosages and approaches. Yet despite similar reviews (e.g.^42,43,44^ also observing these differences, they did not consistently report problematic heterogeneity for these outcomes. One explanation for this contrast is that our review's recency naturally meant that we included additional studies. Some of these studies (e.g.^79,104^ were subsequently identified as outliers, with clear differences observed in the levels of heterogeneity in meta-analyses of for depression (0% vs. 90%), burden (9% vs. 93%) and stress (0% vs. 97%) outcomes’ heterogeneity levels.

Another unifying feature across these and another outlier study^62^ is that they were the only ones to involve scheduled contact with an advisor. Previous reviews have reported improvements to interventions’ effects on outcomes when involving a professional.^44,113^ ND-caregivers value asynchronous electronic communication (e.g. email) with professionals for affording both more accurate documentation of symptom changes^113^ and management of challenging situations (e.g. behavioral disturbances) outside of usual clinical hours.^114^ However, advisors can undertake a variety of roles (e.g. delivering content, moderating web-based forums) and the mechanisms underlying any enhanced efficacy require exploration. The interventionist's confidence in delivering material or support,^115^ or ability to adapt their communication style to web-based formats,^116^ could also be influential.

Intervention features’ heterogeneity also precluded assessment of which were the most efficacious. No approach or mode of delivery afforded within-outcome groups of comparable sizes. Interventions were either based on a select few features (e.g. education k  =  38), or disparate combinations of approaches and modes of delivery, leading to too few cases. The lack of interventions reporting a theoretical basis (27%) similarly impeded quantitative analysis. This lacuna remains a persistent observation across similar reviews (e.g..^41,43,114,129^).

Follow-up data was only reported in five studies, with none reporting measures later than six months from baseline. In fact, only six of the 20 RCT's providing suitable data for analyses reported measures later than three months post baseline, with just one beyond six months (100; at 12 months). It is feasible that certain outcomes, such as quality of life require a longer time-period for significant reductions to manifest.^113^ Furthermore, these outcomes may be intensified by the onset of factors external to the intervention's scope; such as deterioration in patient-symptomology^117^ or increased caregiving-hours.^118^ Conversely, caregivers may not exhibit sufficiently severe symptoms at baseline for an intervention to demonstrate a meaningful effect. Study authors would therefore need to apply any instrument threshold scores that delineate moderate/high levels of an outcome, enabling them to target participants with significant levels of baseline symptoms.

Attrition data was provided in 31 studies, specifically in 17 out of 20 RCTs with usable data. Rates did not appear to be related to use of a supplementary mode (e.g. telephone, email), or any other intervention features. Metrics and measures about actual intervention usage varied across studies, homologous to other reviews (e.g.^45,119^). Low usage and high attrition rates are well documented in internet-facilitated intervention studies.^29,120^ Here, inconsistently reported reasons for drop out inhibited the exploration of possible causes. One possible antecedent; caregivers’ lack of capability with internet-facilitated technology, was mostly unassessed either pre- or post-intervention. Expectations that already-burdened caregivers, whether possessing high or low proficiency in information and communication technology (ICT), master a new platform may be impractical^121^; for it is often incommensurate with patient-monitoring responsibilities.^122^ In absence of any thorough investigation, high attrition is a persistent issue in internet-facilitated technology intervention studies.

### Strengths and limitations

Among this review's strengths is the robust number of RCT's (k  =  26) and studies overall (k  =  51) retrieved through employing a highly sensitive search. Substantial data on the different approaches was accumulated, theoretical bases and design features employed, which were categorized using established classification systems. We were also able to extract sufficient statistical data to conduct meta-analyses for nine separate outcomes. Yet our findings should also be cautiously interpreted on account of the GRADE evidence-quality assessments, which were all very low.

Circumspection is also required when attempting to generalize our findings. By restricting the search to English language studies, relevant studies in other languages may have been missed. Similarly, 45 of the 51 studies were conducted in the USA, Europe or Australia, indicating a paucity of important perspectives from non-Western cultures and low to middle-income countries (LMIC's). For example, Magaña, Martinez &, Loyola's^123^ meta-analysis of caregiving in LMIC's calculated adverse health outcomes for informal caregivers of both South American and Asian, but not African, countries. A more diverse geographical spread in research is clearly needed before attempting to apply extant knowledge or techniques to populations considered previously underrepresented in the literature.

### Future directions

Our meta-analytic calculations for subjective sense of mastery outcomes incorporated solely self-report measures. Future meta-analyses could extend our results by exploring whether interventions achieve commensurate improvements in objective mastery measures. Comparisons of both types have hitherto been examined in narrative,^127^ scoping^129,131^ and systematic reviews.^25,128,130^ Results range from improvements in subjective^127^ or objective only^130^ to both.^25,128,131^ Yet these reviews also exhibit the between-intervention heterogeneity that our review has highlighted. Differences in population features, such as some interventions including formal caregivers, or methodological features, such as comparing both established and intervention-specific outcomes-measures, possibly account for some of these disparate findings.

Other comparisons across studies remain problematic, due to the proliferation of different study designs, quality and outcome measures used. Reportage of population characteristics is similarly heterogeneous, limiting any direct comparisons. Certain sociodemographic variables may be indicative of other factors that influence intervention-usage and efficacy, such as baseline ICT skills, confidence or attitudes toward ICT-usage. Researchers need to not only use established scales to detect these constructs, but also build them into their intervention-implementation, (e.g. through appropriate and consistent technical support).

Level of engagement with the intervention is also salient, although it is often only measured post-intervention using non-validated questionnaires.^124^ Preferably, engagement needs to be clearly defined and continuously assessed throughout testing, using measures which go beyond simple usage-frequency metrics (e.g. dwell-time, components-viewed and completed). By identifying propitious or inhibitory usage components in-situ, rather than waiting for retrospective feedback, it is possible to elucidate the seemingly inevitable tool-usage decline observed at trials’ latter stages.^45^

Since internet-facilitated intervention studies were first published, smartphone ownership has greatly increased, providing access to applications housing multiple functions.^33^ As ICT access for traditionally underserved cohorts continues to expand, the opportunity to better support informal caregivers needs to be explored Intervention design needs to adapt to recent shifts in how individuals favour different devices for different purposes.^125^ Traditionally desktop computer-based activities, such as reading lengthy information studies, or completing multi-item measurement questionnaires, need to be adapted for different size-screens.

## Conclusion

Our comprehensive review demonstrated some support for the efficacy of internet-facilitated interventions in improving sense of mastery and anxiety outcomes for informal caregivers of ND-diagnosed, community-based patients. However, these changes were principally detected after short-term intervention-exposure, with most lasting three months or less. Not only are longer intervention testing-periods required; there remains a paucity of internet-facilitated interventions for caregivers in rare ND-Diagnoses. The growing prevalence of other NDs, such as PD, will undoubtedly engender a concomitant growth in the numbers of informal caregivers. Therefore, practical interventions to safeguard their psychological health is an evident research priority.

## Supplemental Material

sj-docx-1-dhj-10.1177_20552076221129069 - Supplemental material for Internet-facilitated interventions for informal caregivers of patients with neurodegenerative disorders: Systematic review and meta-analysis Click here for additional data file. Supplemental material, sj-docx-1-dhj-10.1177_20552076221129069 for Internet-facilitated interventions for informal caregivers of patients with neurodegenerative disorders: Systematic review and meta-analysis by Neil Boyt, Aileen K Ho, Hannah Morris-Bankole and Jacqueline Sin in Digital Health

sj-docx-2-dhj-10.1177_20552076221129069 - Supplemental material for Internet-facilitated interventions for informal caregivers of patients with neurodegenerative disorders: Systematic review and meta-analysis Click here for additional data file. Supplemental material, sj-docx-2-dhj-10.1177_20552076221129069 for Internet-facilitated interventions for informal caregivers of patients with neurodegenerative disorders: Systematic review and meta-analysis by Neil Boyt, Aileen K Ho, Hannah Morris-Bankole and Jacqueline Sin in Digital Health
