# Supplementary material for: Internet-facilitated interventions for informal caregivers of patients with neurodegenerative disorders: Systematic review and meta-analysis
Source: Digit Health. 2022 Oct 20;8:20552076221129069. doi: 10.1177/20552076221129069 (PMC9585576; doi:10.1177/20552076221129069)
Supplement: sj-docx-2-dhj-10.1177_20552076221129069 - Supplemental material for Internet-facilitated interventions for informal caregivers of patients with neurodegenerative disorders: Systematic review and meta-analysis [file sj-docx-2-dhj-10.1177_20552076221129069.docx]

**APPENDIX 2: SEARCH TERMS BY DATABASE**

**MEDLINE**

Search (((((Mentoring OR "virtual community" OR "online community" OR "mutual support" or "peer support" OR "group support" OR "Social Support" OR "psychosocial support" OR "professional support" OR "self-management" OR "psychological Feedback" OR "Problem solving" OR "health promotion" OR Psychotherapy OR "therapy, computer-assisted" OR therapy OR Psychoeducation*)) AND ( medline[sb] AND "open access"[filter] ) AND ( "1999/01/01"[PDat] : "2021/12/31"[PDat] ))) AND (((((("information communication technology" OR "information technology" OR Videoconferencing OR telecare OR telemonitoring OR Internet OR website* OR "computer-tailored" OR "computer-based" OR "web portal" OR "computer-assisted instruction" OR "online decision aid tools" OR "user-computer interface" OR "web tool" OR digital OR "virtual reality" OR "Web-based")) AND ( medline[sb] AND "open access"[filter] ) AND ( "1999/01/01"[PDat] : "2021/12/31"[PDat] ))) AND ((((((iphone OR smartphone OR "smart phone" OR android OR "cell phone" OR mobile OR email OR "electronic mail" OR "social network*" OR YouTube OR Twitter OR wechat OR "Mobile Applications" OR "social media" OR "mobile apps" OR "Computers, Handheld" OR ipad OR "self-help devices")) AND ( medline[sb] AND "open access"[filter] ) AND ( "1999/01/01"[PDat] : "2021/12/31"[PDat] ))) AND (((((("online system" OR "search engine" OR google OR "artificial intelligence" OR telehealth OR "Medical Informatics" OR "reminder systems" OR mhealth OR "mobile health" OR ehealth OR e-health OR e-learning OR electronic)) AND ( medline[sb] AND "open access"[filter] ) AND ( "1999/01/01"[PDat] : "2021/12/31"[PDat] ))) AND (((("informal caregiv*"[All Fields] OR "informal carer*"[All Fields] OR "caregiv*"[All Fields] OR "carer*"[All Fields] OR "care giving"[All Fields] OR "family"[MeSH Terms] OR "family"[All Fields] OR "familial"[All Fields] OR "families"[All Fields] OR "family s"[All Fields] OR "familys"[All Fields] OR "couple*"[All Fields] OR "partner*"[All Fields] OR "spous*"[All Fields] OR "spouses"[MeSH Terms] OR "spouses"[All Fields] OR "wife"[All Fields] OR "husband*"[All Fields] OR "parent*"[All Fields] OR "stepparent*"[All Fields] OR "grandparent*"[All Fields] OR "cohabit"[All Fields] OR "cohabitant"[All Fields] OR "cohabitants"[All Fields] OR "cohabitate"[All Fields] OR "cohabitated"[All Fields] OR "cohabitating"[All Fields] OR "cohabitations"[All Fields] OR "cohabiter"[All Fields] OR "cohabiters"[All Fields] OR "cohabiting"[All Fields] OR "cohabits"[All Fields] OR "child*"[All Fields] OR "friend s"[All Fields] OR "friending"[All Fields] OR "friends"[MeSH Terms] OR "friends"[All Fields] OR "friend"[All Fields] OR "volunteer*"[All Fields] OR "companion s"[All Fields] OR "friends"[MeSH Terms] OR "friends"[All Fields] OR "companion"[All Fields] OR "companions"[All Fields]) AND ("parkinsons disease"[All Fields] OR "huntingtons disease"[All Fields] OR "motor neuron disease"[All Fields] OR "amyotrophic lateral sclerosis"[All Fields] OR "Alzheimers Disease"[All Fields] OR "Lewy Body Dementia"[All Fields] OR "dementia"[MeSH Terms] OR "dementia"[All Fields] OR "dementias"[All Fields] OR "dementia s"[All Fields] OR "vascular disease"[All Fields] OR "Multiple Sclerosis"[All Fields] OR "neurodegenerative disease*"[All Fields]))) AND ( medline[sb] AND "open access"[filter] ) AND ( "1999/01/01"[PDat] : "2021/12/31"[PDat] ))) AND ( medline[sb] AND "open access"[filter] ) AND ( "1999/01/01"[PDat] : "2021/12/31"[PDat] ))) AND ( medline[sb] AND "open access"[filter] ) AND ( "1999/01/01"[PDat] : "2021/12/31"[PDat] ))) AND ( medline[sb] AND "open access"[filter] ) AND ( "1999/01/01"[PDat] : "2021/12/31"[PDat] ))) Filters: MEDLINE journals Sort by: PubDate

**MEDLINE in-process (via Ovid)**

**1** ((((((((("neurodegenerative disease*") OR "Alzheimers disease") OR "huntingtons disease") OR "parkinsons disease") OR "Multiple Sclerosis") OR "motor neuron disease") OR "amyotrophic lateral sclerosis") OR "Lewy body dementia") OR dementia*) OR "vascular disease") *Indexes=MEDLINE Timespan=All years*

2 ((((((((((((((((((("informal caregiv*") OR Caregiv*) OR carer*) OR "care giving") OR famil*) OR grandparent*) OR Couple*) OR Partner*) OR Spous*) OR wife) OR Husband*) OR marri*) OR parent*) OR stepparent*) OR cohabitat*) OR child*) OR friend) OR volunteer*) OR "care provider") OR companion) *Indexes=MEDLINE*

3 ((((((((((((((((((((((((((((((((((((((((((((("online system") OR "search engine") OR google) OR "artificial intelligence") OR telehealth) OR telemedicine) OR "Medical Informatics") OR "reminder systems") OR mhealth) OR "mobile health") OR ehealth) OR e-health) OR e-learning) OR electronic) OR iphone) OR smartphone) OR "smart phone") OR android) OR mobile) OR "cell phone") OR email) OR "social network*") OR YouTube) OR Twitter) OR wechat) OR "Mobile Applications") OR "social media") OR "mobile apps") OR "Computers, Handheld") OR ipad) OR "self-help devices") OR Videoconferencing) OR telecare) OR telemonitoring) OR Internet) OR website*) OR "computer-tailored") OR "computer-based") OR "web portal") OR "computer-assisted instruction") OR "online decision aid tools") OR "user-computer interface") OR "web tool") OR digital) OR "virtual reality") OR ' "Web-based")

4(((((((((((((((Mentoring) OR "virtual community") OR "online community") OR "mutual support") OR "peer support") OR "group support") OR "Social Support") OR "psychosocial support") OR "professional support") OR "self-management") OR "psychological Feedback") OR "Problem solving") OR "health promotion") OR Psychotherap*) OR "therapy, computer-assisted") OR Psychoeducation*)

**NIH Clinical Trials**

"online system" OR "search engine" OR google OR "artificial intelligence" OR telehealth OR "Medical Informatics" OR "reminder systems" OR mhealth OR "mobile health" OR ehealth OR e-health OR e-learning OR electronic OR iphone OR smartphone AND caregiver AND caregiver | Active, not recruiting, Completed Studies | Interventional Studies | "parkinsons disease" OR "huntingtons disease" OR "motor neuron disease" OR "amyotrophic lateral sclerosis" OR "Alzheimers Disease" OR "Lewy Body Dementia" OR dementia OR "vascular disease" OR "Multiple Sclerosis" OR "neurodegenerative disease*" | Adult, Older Adult

**PubMed**

(("mentor s"[All Fields] OR "mentored"[All Fields] OR "mentoring"[MeSH Terms] OR "mentoring"[All Fields] OR "mentors"[MeSH Terms] OR "mentors"[All Fields] OR "mentor"[All Fields] OR "virtual community"[All Fields] OR "online community"[All Fields] OR "mutual support"[All Fields] OR "peer support"[All Fields] OR "group support"[All Fields] OR "Social Support"[All Fields] OR "psychosocial support"[All Fields] OR "professional support"[All Fields] OR "self-management"[All Fields] OR "psychological Feedback"[All Fields] OR "Problem solving"[All Fields] OR "health promotion"[All Fields] OR ("psychotherapie"[All Fields] OR "psychotherapy"[MeSH Terms] OR "psychotherapy"[All Fields] OR "psychotherapies"[All Fields] OR "psychotherapy s"[All Fields]) OR "therapy computer assisted"[All Fields] OR ("therapeutics"[MeSH Terms] OR "therapeutics"[All Fields] OR "therapies"[All Fields] OR "therapy"[MeSH Subheading] OR "therapy"[All Fields] OR "therapy s"[All Fields] OR "therapys"[All Fields]) OR "psychoeducation*"[All Fields]) AND "english"[Language] AND (("online system"[All Fields] OR "search engine"[All Fields] OR ("google"[All Fields] OR "google s"[All Fields] OR "googled"[All Fields] OR "googling"[All Fields]) OR "artificial intelligence"[All Fields] OR ("telehealth s"[All Fields] OR "telemedicine"[MeSH Terms] OR "telemedicine"[All Fields] OR "telehealth"[All Fields]) OR "Medical Informatics"[All Fields] OR "reminder systems"[All Fields] OR ("mhealth s"[All Fields] OR "telemedicine"[MeSH Terms] OR "telemedicine"[All Fields] OR "mhealth"[All Fields]) OR "mobile health"[All Fields] OR ("telemedicine"[MeSH Terms] OR "telemedicine"[All Fields] OR "ehealth"[All Fields]) OR "e-health"[All Fields] OR "e-learning"[All Fields] OR ("electronical"[All Fields] OR "electronically"[All Fields] OR "electronics"[MeSH Terms] OR "electronics"[All Fields] OR "electronic"[All Fields]) OR ("iphone"[All Fields] OR "iphones"[All Fields]) OR ("smartphone"[MeSH Terms] OR "smartphone"[All Fields] OR "smartphones"[All Fields] OR "smartphone s"[All Fields]) OR "smart phone"[All Fields] OR "android"[All Fields]) OR "cell phone"[All Fields] OR ("mobile"[All Fields] OR "mobiles"[All Fields]) OR ("electronic mail"[MeSH Terms] OR ("electronic"[All Fields] AND "mail"[All Fields]) OR "electronic mail"[All Fields] OR "email"[All Fields] OR "emails"[All Fields] OR "emailed"[All Fields] OR "emailing"[All Fields]) OR "electronic mail"[All Fields] OR "social network*"[All Fields] OR ("youtube"[All Fields] OR "youtube s"[All Fields]) OR ("twitter"[All Fields] OR "twitter s"[All Fields] OR "twitters"[All Fields]) OR "wechat"[All Fields] OR "Mobile Applications"[All Fields] OR "social media"[All Fields] OR "mobile apps"[All Fields] OR "computers handheld"[All Fields] OR "ipad"[All Fields] OR "self-help devices"[All Fields] OR "information communication technology"[All Fields] OR ("informaton"[All Fields] AND ("technology"[MeSH Terms] OR "technology"[All Fields] OR "technologies"[All Fields] OR "technology s"[All Fields])) OR ("videoconferenced"[All Fields] OR "videoconferencing"[MeSH Terms] OR "videoconferencing"[All Fields]) OR "telecare"[All Fields] OR ("telemonitor"[All Fields] OR "telemonitored"[All Fields] OR "telemonitoring"[All Fields] OR "telemonitors"[All Fields]) OR ("internet"[MeSH Terms] OR "internet"[All Fields] OR "internet s"[All Fields] OR "internets"[All Fields]) OR "website*"[All Fields] OR "computer-tailored"[All Fields] OR "computer-based"[All Fields] OR "web portal"[All Fields] OR "computer-assisted instruction"[All Fields] OR ("online"[All Fields] AND ("decision support techniques"[MeSH Terms] OR ("decision"[All Fields] AND "support"[All Fields] AND "techniques"[All Fields]) OR "decision support techniques"[All Fields] OR ("decision"[All Fields] AND "aid"[All Fields]) OR "decision aid"[All Fields]) AND ("tool s"[All Fields] OR "tools"[All Fields])) OR "user-computer interface"[All Fields] OR "web tool"[All Fields] OR ("digital"[All Fields] OR "digitalisation"[All Fields] OR "digitalised"[All Fields] OR "digitalization"[All Fields] OR "digitalize"[All Fields] OR "digitalized"[All Fields] OR "digitalizer"[All Fields] OR "digitalizing"[All Fields] OR "digitally"[All Fields] OR "digitals"[All Fields] OR "digitization"[All Fields] OR "digitizations"[All Fields] OR "digitize"[All Fields] OR "digitized"[All Fields] OR "digitizer"[All Fields] OR "digitizers"[All Fields] OR "digitizes"[All Fields] OR "digitizing"[All Fields]) OR "virtual reality"[All Fields] OR "Web-based"[All Fields]) AND "english"[Language]) AND ("informal caregiv*"[All Fields] OR "informal carer*"[All Fields] OR "caregiv*"[All Fields] OR "carer*"[All Fields] OR "care giving"[All Fields] OR ("familialities"[All Fields] OR "familiality"[All Fields] OR "familially"[All Fields] OR "familials"[All Fields] OR "familie"[All Fields] OR "family"[MeSH Terms] OR "family"[All Fields] OR "familial"[All Fields] OR "families"[All Fields] OR "family s"[All Fields] OR "familys"[All Fields]) OR "relative*"[All Fields] OR "couple*"[All Fields] OR "partner*"[All Fields] OR "spous*"[All Fields] OR ("spouses"[MeSH Terms] OR "spouses"[All Fields] OR "wife"[All Fields]) OR "husband*"[All Fields] OR "marri*"[All Fields] OR "parent*"[All Fields] OR "stepparent*"[All Fields] OR "grandparent*"[All Fields] OR ("cohabit"[All Fields] OR "cohabitant"[All Fields] OR "cohabitants"[All Fields] OR "cohabitate"[All Fields] OR "cohabitated"[All Fields] OR "cohabitating"[All Fields] OR "cohabitation"[All Fields] OR "cohabitational"[All Fields] OR "cohabitations"[All Fields] OR "cohabited"[All Fields] OR "cohabiter"[All Fields] OR "cohabiters"[All Fields] OR "cohabiting"[All Fields] OR "cohabits"[All Fields]) OR "child*"[All Fields] OR ("friend s"[All Fields] OR "friending"[All Fields] OR "friends"[MeSH Terms] OR "friends"[All Fields] OR "friend"[All Fields]) OR "volunteer*"[All Fields] OR ("companion s"[All Fields] OR "friends"[MeSH Terms] OR "friends"[All Fields] OR "companion"[All Fields] OR "companions"[All Fields])) AND ("parkinsons disease"[All Fields] OR "huntingtons disease"[All Fields] OR "motor neuron disease"[All Fields] OR "amyotrophic lateral sclerosis"[All Fields] OR "Alzheimers Disease"[All Fields] OR "Lewy Body Dementia"[All Fields] OR ("dementia"[MeSH Terms] OR "dementia"[All Fields] OR "dementias"[All Fields] OR "dementia s"[All Fields]) OR "vascular disease"[All Fields] OR "Multiple Sclerosis"[All Fields] OR "neurodegenerative disease*"[All Fields])) AND (english[Filter])

**PsycINFO (via Ovid)**

| **#** | **Query** |
| --- | --- |
| 1 | (neurodegenerative disease* or Alzheimers disease or motor neuron disease or Lewy body dementia or vascular disease or Multiple Sclerosis or dementia* or amyotrophic lateral sclerosis or parkinsons disease or huntingtons disease).mp. [mp=title, abstract, heading word, table of contents, key concepts, original title, tests & measures, mesh] |
| 2 | (informal caregiv* or Caregiv* or carer* or care giving or famil* or Couple* or Partner* or Spous* or wife or Husband* or marri* or parent* or stepparent* or cohabitat* or child* or friend or volunteer* or care provider or companion or grandparent).mp. [mp=title, abstract, heading word, table of contents, key concepts, original title, tests & measures, mesh] |
| 3 | (Mentoring or virtual community or online community or mutual support or peer support or group support or Social Support or psychosocial support or professional support or self-management or psychological Feedback or Problem solving or health promotion or Psychotherap* or therap*, computer-assisted or Psychoeducation*).mp. [mp=title, abstract, heading word, table of contents, key concepts, original title, tests & measures, mesh] |
| 4 | (information communication technology or information technology or Videoconferencing or telecare or telemonitoring or Internet or website* or computer-tailored or computer-based or web portal or computer-assisted instruction or online decision aid tools or user-computer interface or web tool or digital or virtual reality or Web-based or iphone or smartphone or smart phone or android or cell phone* or mobile or email or electronic mail or social network* or YouTube or Twitter or wechat or Mobile Applications or social media or mobile apps or Computers, Handheld or ipad or self-help devices or online system or search engine or google or artificial intelligence or telehealth or telemedicine or medical Informatics or reminder systems or mhealth or mobile health or ehealth or e-health or e-learning or electronic).mp. [mp=title, abstract, heading word, table of contents, key concepts, original title, tests & measures, mesh] |
| 5 | 1 and 2 and 3 and 4 |

**Web of Science**

**#1 TOPIC:** ("neurodegenerative disease*" OR "Alzheimers disease" OR "huntingtons disease" OR "parkinsons disease" OR "Multiple Sclerosis" OR "motor neuron disease" OR "amyotrophic lateral sclerosis" OR "Lewy body dementia" OR dementia* OR "vascular disease")

*Indexes=SCI-EXPANDED, SSCI, A&HCI, CPCI-S, CPCI-SSH, BKCI-S, BKCI-SSH, ESCI, CCR-EXPANDED, IC Timespan=All years*

#2 **TOPIC:**  ("informal caregiv*" OR Caregiv* OR carer* OR "care giving" OR famil* OR grandparent* OR Couple* OR Partner* OR Spous* OR wife OR Husband* OR marri* OR parent* OR stepparent* OR cohabitat* OR child* OR friend OR volunteer* OR "care provider" OR companion)

*Indexes=SCI-EXPANDED, SSCI, A&HCI, CPCI-S, CPCI-SSH, BKCI-S, BKCI-SSH, ESCI, CCR-EXPANDED, IC Timespan=All years*

#3 **TOPIC:** ("online system" OR "search engine" OR google OR "artificial intelligence" OR telehealth OR telemedicine OR "Medical Informatics" OR "reminder systems" OR mhealth OR "mobile health" OR ehealth OR e-health OR e-learning OR electronic OR iphone OR smartphone OR "smart phone" OR android OR mobile OR "cell phone" OR email OR "social network*" OR YouTube OR Twitter OR wechat OR "Mobile Applications" OR "social media" OR "mobile apps" OR "Computers, Handheld" OR ipad OR "self-help devices" OR Videoconferencing OR telecare OR telemonitoring OR Internet OR website* OR "computer-tailored" OR "computer-based" OR "web portal" OR "computer-assisted instruction" OR "online decision aid tools" OR "user-computer interface" OR "web tool" OR digital OR "virtual reality" OR ' "Web-based")

*Indexes=SCI-EXPANDED, SSCI, A&HCI, CPCI-S, CPCI-SSH, BKCI-S, BKCI-SSH, ESCI, CCR-EXPANDED, IC Timespan=All years*

#4 **TOPIC:** (Mentoring OR "virtual community" OR "online community" OR "mutual support" OR "peer support" OR "group support" OR "Social Support" OR "psychosocial support" OR "professional support" OR "self-management" OR "psychological Feedback" OR "Problem solving" OR "health promotion" OR Psychotherap* OR "therapy, computer-assisted" OR Psychoeducation*)

*Indexes=SCI-EXPANDED, SSCI, A&HCI, CPCI-S, CPCI-SSH, BKCI-S, BKCI-SSH, ESCI, CCR-EXPANDED, IC Timespan=All years*

#5 #1 AND #2 AND #3 AND #4 AND #5

**Cochrane Central Register of Controlled Trials (CENTRAL)**

#1 "parkinsons disease" OR “huntingtons disease” OR “motor neuron disease” OR amyotrophic lateral sclerosis OR "Alzheimers Disease" OR "Lewy Body Dementia" OR dementia OR "vascular disease" OR "Multiple Sclerosis" OR “neurodegenerative disease*”

#2 “informal caregiver” OR “informal caregiving” OR Caregiv* OR carer* OR “care giving” OR family OR relative* OR Couple* OR Partner* OR Spous* OR wife OR Husband* OR marri* OR parent* OR stepparent* OR grandparent* OR cohabitant OR child* OR friend OR volunteer* OR companion

#3 "online system" OR "search engine" OR google OR "artificial intelligence" OR telehealth OR "Medical Informatics" OR "reminder systems" OR mhealth OR "mobile health" OR ehealth OR e-health OR e-learning OR electronic OR iphone OR smartphone OR "smart phone" OR android OR mobile OR email OR "social network*" OR YouTube OR Twitter OR wechat OR "Mobile Applications" OR "social media" OR "mobile apps" OR "Computers, Handheld" OR ipad OR "self-help devices" OR technology OR Videoconferencing OR telecare OR telemonitoring OR Internet OR website* OR "computer-tailored" OR "computer-based" OR "web portal" OR "computer-assisted instruction" OR "online decision aid tools" OR "user-computer interface" OR "web tool" OR digital OR "virtual reality" OR "Web-based" with Publication Year from 2000 to 2020, in Trials

#4 Mentoring OR "virtual community" OR "online community" OR "mutual support" or "peer support" OR "group support" OR "Social Support" OR "psychosocial support" OR "professional support" OR "self-management" OR "psychological Feedback" OR "Problem solving" OR "health promotion" OR Psychotherapy OR "therapy, computer-assisted" OR therapy OR Psychoeducation*

#5 #1 AND #2 AND #3 AND #4

**APPENDIX 3: EXCLUDED STUDIES CLASSIFIED AS ‘NEAR MISSES’**

| STUDY REFERENCE | EXCLUSION REASON |
| --- | --- |
|  |  |
| Adamski, T & Alfaro, M W (2009) Virtual psycho-educative support groups for caregivers of persons diagnosed with dementia. Caring : National Association for Home Care magazine 28(8) 44-6 | Outcome-measures criteria not met |
| Banbury, A ;Parkinson, L ;Gordon, S ;Wood, D (2019) Implementing a peer-support programme by group videoconferencing for isolated carers of people with dementia Journal of Telemedicine and Telecare 25(9) 572-577 | Outcome-measures criteria not met |
| Barbabella F, Poli A, Andréasson F, Salzmann B, Papa R, Hanson E, Efthymiou A, Döhner H, Lancioni C, Civerchia P & Lamura G (2016) A Web-Based Psychosocial Intervention for Family Caregivers of Older People: Results from a Mixed-Methods Study in Three European Countries JMIR Res Protoc 5(4):e196 DOI: 10.2196/resprot.5847 | Not exclusively ND patients |
| Bartels SL, . van Knippenberg R. J. M, Köhler S., Ponds R. W. ,Myin-Germeys I, Verhey F. R. J.& de Vugt M. E.(2019) The necessity for sustainable intervention effects: lessons-learned from an experience sampling intervention for spousal carers of people with dementia, Aging & Mental Health, DOI: 10.1080/13607863.2019.1647130 cf. cf. van Knippenberg, R. J. M.; de Vugt, M. E.; Ponds, R. W.; et al. (2018) An Experience Sampling Method Intervention for Dementia Caregivers: Results of a Randomized Controlled Trial AMERICAN JOURNAL OF GERIATRIC PSYCHIATRY 26(12) 1231-46 | <50% of intervention delivery through internet |
| Beauchamp, N. ;Irvine, A. B. ;Seeley, J. & Johnson, B. (2005) Worksite-based internet multimedia program for family caregivers of persons with dementia Gerontologist 45(6) 793-801 | Non-informal caregivers |
| BOETTCHE, M.; KLASEN, M. ; KNAEVELSRUD, CHR (2013) AN INTERNET-BASED INTERVENTION FOR HEALTH PROMOTION IN FAMILY CAREGIVERS - RESULTS OF A PILOT STUDY PSYCHIATRISCHE PRAXIS 40(6) 327-331 DOI http://dx.doi.org/ 10.1055/s-0033-134950 | Not exclusively ND patients |
| Boyd, K. ;Nugent, C. ;Donnelly, M. ;Bond, R. ;Sterritt, R. & Hartin, P. (2014) An investigation into the usability of the STAR training and re-skilling website for carers of persons with dementia Year: Journal:Conf Proc IEEE Eng Med Biol Soc 2014 4139-42 | Review or usability study only |
| Brody, A. A. ;Guan, C. ;Cortes, T. ;Galvin, J. E. (2016) Development and testing of the Dementia Symptom Management at Home (DSM-H) program: An interprofessional home health care intervention to improve the quality of life for persons with dementia and their caregivers Geriatr Nurs 37(3) 200-6 | Non-informal caregivers |
| Brown, E. L., Ruggiano, N., Page, T. F., Roberts, L., Hristidis, V., Whiteman, K. L., & Castro, J. (2016). CareHeroes Web and Android™ Apps for Dementia Caregivers: A Feasibility Study. Research in gerontological nursing, 9(4), 193–203. https://doi.org/10.3928/19404921-20160229-02 | Non-caregiver focused intervention or measures |
| Butcher HK, Gordon JK, Ko JW, et al. (2016) Finding Meaning in Written Emotional Expression by Family Caregivers of Persons With Dementia. Am J Alzheimers Dis Other Demen. 31(8):631-642. doi:10.1177/1533317516660611 | <50% of intervention delivery through internet |
| Callan, J.A., Siegle, G.J., Abebe, K., Black, B., Martire, L., Schulz, R., Reynolds, C. & Hall, M.H., (2016) Feasibility of a pocket-PC based cognitive control intervention in dementia spousal caregivers. Aging & Mental Health.. doi:10.1080/13607863.2015.1031635 | Outcome-measures criteria not met |
| Czaja, Sara, PhD, and, Rubert, Mark. (2002) Telecommunications Technology as an Aid to Family Caregivers of Persons With Dementia. Psychosom Med. 64(3): 469-476. | Review or usability study only |
| Davis, B.H. ;Shehab, M. ;Shenk, D. & Nies, M (2015) E-mobile pilot for community-based dementia caregivers identifies desire for security Gerontechnology 13(3) 332-336 | Non-informal caregivers |
| Eisdorfer, C. ;Czaja, S. J. ;Loewenstein, D. A. ;Rubert, M. P. ;Arguelles, S. ;Mitrani, V. B. & Szapocznik, J. (2003) The effect of a family therapy and technology-based intervention on caregiver depression Gerontologist 43 (4) 521-531 | <50% of intervention delivery through internet |
| Gately, ME; Trudeau, SA & Moo, LR (2020) Feasibility of Telehealth-Delivered Home Safety Evaluations for Caregivers of Clients With Dementia OTJR-OCCUPATION PARTICIPATION AND HEALTH 40 (1) 42-49 DOI: 10.1177/1539449219859935 | Non-caregiver focused intervention or measures |
| Gaugler, Joseph E. ;Reese, Mark ;Tanler, Richard (2016) Care to Plan: An Online Tool That Offers Tailored Support to Dementia Caregivers Gerontologist 56(6) 1161-1174 | Non-community based patient or intervention |
| GLUECKAUF R. L., KETTERSON T. U., LOOMIS J. S, & DAGES P. (2004) Online Support and Education for Dementia Caregivers: Overview, Utilization, and Initial Program Evaluation TELEMEDICINE JOURNAL AND e-HEALTH 10(2) | <50% of intervention delivery through internet |
| Han, J., Guo, G., & Hong, L. (2020) Impact of professionally facilitated peer support for family carers of people with dementia in a WeChat virtual community. Journal of Telemedicine and Telecare. https://doi.org/10.1177/1357633X20910830 | Non-community based patient or intervention |
| Hattink B. J. J.,. Meiland F. J. M, Overmars-Marx T. ,de Boer M., Ebben P. W. G., van Blanken M., Verhaeghe S. , Stalpers-Croeze, Jedlitschka I.A., Flick S. E., v/d Leeuw J., Karkowski I. & Dröes R. M. (2016) The electronic, personalizable Rosetta system for dementia care: exploring the user-friendliness, usefulness and impact, Disability and Rehabilitation: Assistive Technology, 11(1), 61-71, DOI: 10.3109/17483107.2014.932022 | Not exclusively ND patients |
| Hayden, LJ ; Glynn, SM ; Hahn, TJ ; Randall, F & Randolph, E (2012) The Use of Internet Technology for Psychoeducation and Support With Dementia Caregivers PSYCHOLOGICAL SERVICES 9(2) 215-218 Special Issue: SI DOI: 10.1037/a0027056 | <50% of intervention delivery through internet |
| Kajiyama, B.K, Dib, L. R. C, Tymchuk, A.r J, Boxer, A. L, Kixmiller, J. S & Olinsky, C. J. (2007) Improving the quality of life for caregivers and care recipients with personalized video channels. Clinical Gerontologist: The Journal of Aging and Mental Health, 31, 95-100. https://doi.org/10.1300/J018v31n01_07 | Non caregiver-focused intervention or outcome measures |
| Lewis, ML ; Hobday, JV & Hepburn, KW (2010) Internet-Based Program for Dementia Caregivers AMERICAN JOURNAL OF ALZHEIMERS DISEASE AND OTHER DEMENTIAS 25(8) 674-679  DOI: 10.1177/1533317510385812 | Outcome-measures criteria not met |
| Lorenz, K, Freddolino, P P, Comas-Herrera, A, Knapp, M & Damant, J. (2019) Technology-based tools and services for people with dementia and carers: Mapping technology onto the dementia care pathway. Dementia: The International Journal of Social Research and Practice, 18, 725-741. https://doi.org/10.1177/1471301217691617 | Review or usability study only |
| Lorig, K ;Thompson-Gallagher, D ;Traylor, L ;Ritter, P L. ;Laurent, DD. ;Plant, K ;Thompson, L W. & Hahn, TJ. (2012) Building Better Caregivers: A Pilot Online Support Workshop for Family Caregivers of Cognitively Impaired Adults :Journal of Applied Gerontology 31(3) 423-437 | Not exclusively ND patients |
| M. Lyu & S. Yuan, (2020) "Cloud-Based Smart Dog Music Therapy and Pneumonia Detection System for Reducing the Difficulty of Caring for Patients With Dementia," in IEEE Access, 8 20977-20990, , doi: 10.1109/ACCESS.2020.2969482 | Outcome-measures criteria not met |
| Marziali, E ;Donahue, P & Crossin, G (2005) Caring for Others: Internet Health Care Support Intervention for Family Caregivers of Persons With Alzheimer's, Stroke, or Parkinson's Disease Families in Society Volume: 86(3) 375-383 | Outcome-measures criteria not met |
| Marziali, E ;Damianakis, T & Donahue, P (2006) Internet-based clinical services: Virtual support groups for family caregivers Journal of Technology in Human Services 24(2-3) 39-54 | Outcome-measures criteria not met |
| R. F. Navarro, M. D. Rodríguez and J. Favela, (2014) Intervention Tailoring in Augmented Cognition Systems for Elders With Dementia," in IEEE Journal of Biomedical and Health Informatics, 18(1) 361-367 doi: 10.1109/JBHI.2013.2267542. | Non caregiver-focused intervention or outcome measures |
| Possin, KL ; Merrilees, JJ ; Dulaney, S ; Bonasera, SJ ; Chiong, W ; Lee, K; Hooper, SM ; Allen, IE ; Braley, T ; Bernstein, A; Rosa, TD ; Harrison, K ; Begert-Hellings, H; Kornak, J; Kahn, JG; Naasan, G; Lanata, S; Clark, AM; Chodos, A ; Gearhart, R ; Ritchie, C & Miller, BL(2019) Effect of Collaborative Dementia Care via Telephone and Internet on Quality of Life, Caregiver Well-being, and Health Care Use The Care Ecosystem Randomized Clinical Trial JAMA INTERNAL MEDICINE 179(12) 1658-1667 DOI: 10.1001/jamainternmed.2019.4101 | <50% of intervention delivery through internet |
| Pot, AM; Blom, MM & Willemse, BM (2015) Acceptability of a guided self-help Internet intervention for family caregivers: mastery over dementia INTERNATIONAL PSYCHOGERIATRICS 27(8) 1343-1354 DOI: 10.1017/S1041610215000034 | Non-community based patient or intervention |
| Reichert, M., Hampel, S. & Reuter, V. (2016) Mobile dementia counseling as a low-threshold offer of help for caring relatives. Z Gerontol Geriat 49, 181-186 . https://doi.org/10.1007/s00391-016-1029-x | <50% of intervention delivery through internet |
| Rutz, M ; Gerlach, M ; Schmeer, R ; Gaugisch, P ; Bauer, A ; Wolff, D; Behrends, M; Kupka, T; Raudies, S; Meyenburg-Altwarg, I (2019) Providing knowledge and support for caring relatives with the smartphone - the MoCaB project 32(6) 305-314 Special Issue: SI DOI: 10.1024/1012-5302/a000695 | Review or usability study only |
| Schaller, S., Marinova-Schmidt, V., Gobin, J., Criegee-Rieck, M., Griebel, L., Engel, S., Stein, V., Graessel, E., Kolominsky-Rabas, P.L. (2015) Tailored e-Health services for the dementia care setting: a pilot study of ‘eHealthMonitor’. BMC Medical Informatics and Decision Making.. doi:10.1186/s12911-015-0182-2 | Outcome-measures criteria not met |
| Steffen, A.M. & Gant, J.R. (2016) A telehealth behavioral coaching intervention for neurocognitive disorder family carers. International Journal of Geriatric Psychiatry doi:10.1002/gps.4312 | <50% of intervention delivery through internet |
| Torp, S., Hanson, E., Hauge, S., Ulstein, I. & Magnusson, L (2007) A pilot study of how information and communication technology may contribute to health promotion among elderly spousal carers in Norway. Health & Social Care in the Community.. doi:10.1111/j.1365-2524.2007.00725.x | Not exclusively ND patients |
| Torp, S; Bing-Jonsson, PC & Hanson, E (2013) Experiences with using information and communication technology to build a multi-municipal support network for informal carers INFORMATICS FOR HEALTH & SOCIAL CARE 38(3) 265-279 DOI:10.3109/17538157.2012.735733 | Not exclusively ND patients |
| Tyack, C., Camic, P.M., Heron, M.J. & Hulbert, S., (2017). Viewing Art on a Tablet Computer: A Well-Being Intervention for People With Dementia and Their Caregivers. Journal of Applied Gerontology.. doi:10.1177/0733464815617287 | Non caregiver-focused intervention or outcome measures |
| Williams, K; Arthur, A; Niedens, M; Moushey, L & Hutfles, L (2013) In-Home Monitoring Support for Dementia Caregivers: A Feasibility Study CLINICAL NURSING RESEARCH 22(2) 139-150 DOI: 10.1177/1054773812460545 | <50% of intervention delivery through internet |

**APPENDIX 4: TEMPLATE DATA COLLECTION FORM**

| Study Lead Author, Country, ND | Chi&Demiris (2015) Approach | Webb et al. (2010) Use of Theory | Webb et al. (2010) Mode of Delivery | Facilitator Role Description & Pre- Intervention Training | Study Duration, Design & Delivery Features | Intervention Length, Intensity & Treatment received Individually/ Dyadically/ Group? | Participant Allocation Intervention /Control | Control Arm Type | Outcome Measures Timepoints & Instruments | Caregiver Age (Mean)[SD] Intervention /Control /Total | Caregiver Gender (%) Intervention /Control /Total | Caregiver Relationship to Care- Recipient (%) Intervention /Control /Total | Duration of Caregiving (Mean)[SD] Intervention /Control /Total | Other Reported Caregiver Information Intervention /Control /Total |
| --- | --- | --- | --- | --- | --- | --- | --- | --- | --- | --- | --- | --- | --- | --- |
|  |  |  |  |  |  |  |  |  |  |  |  |  |  |  |
|  |  |  |  |  |  |  |  |  |  |  |  |  |  |  |
|  |  |  |  |  |  |  |  |  |  |  |  |  |  |  |
|  |  |  |  |  |  |  |  |  |  |  |  |  |  |  |
